# Graph theory methods for analyzing functional connectivity in multiple spike trains: application to data recorded from the visual cortex of a cat

**DOI:** 10.1007/s11571-025-10333-y

**Published:** 2025-10-03

**Authors:** Mohammad Shahed Masud, Danko Nikolić, Liz Stuart, Roman Borisyuk

**Affiliations:** 1 Institute of Statistical Research and Training (ISRT), University of Dhaka, Dhaka, Bangladesh; 2 “Robots Go Mental” Company, Frankfurt, Germany; 3 School of Computing, Ulster University, Belfast, Northern Ireland; 4 Department of Mathematics and Statistics, University of Exeter, Exeter, UK

**Keywords:** Simultaneous recordings, Multiple spike trains, Functional connectivity, Cox method, Visual stimulation and response, Graph theory measures

## Abstract

This study explores graph theory methods for analyzing the functional connectivity of multiple spike trains. We study simultaneously recorded multiple spike trains recorded from the visual cortex of a cat under different visual stimuli. To find the functional connectivity for a given visual stimulus we use the Cox method (Masud and Borisyuk, J Neurosci Methods 196:201–219, 2011). The application of graph theory methods for analysing the connectivity circuit, revealed that the functional connectivity of multiple spike trains is characterized by low density, long communication distances, and weak interconnectivity. Nevertheless, some spike trains also exhibit high degrees of centrality, including betweenness centrality, expansiveness coefficient, and attractiveness coefficient. Additionally, the analysis also identified significant motifs within the functional connections. Thus, our approach allows to describe the correspondence between the stimulus and functional connectivity diagram and compare functional connections under different stimuli.

## Introduction

In recent years, technological advancements have led to an increase in the availability of datasets containing simultaneously recorded multiple spike trains from various brain areas (Stevenson and Kording 2011; Buzsáki and Mizuseki 2014; Jun et al. 2017; Yger et al. 2018; Schneider et al. 2018). This abundance of data necessitates the development of appropriate computational tools for comprehensive analysis (Gerhard et al. 2011; Pisarchik 2024). One such analysis tool is graph theory, a branch of mathematics with applications in diverse fields such as physics, communication science, genetics, linguistics, and sociology (Sporns 2013; Badwaik 2020). Over the past decade, graph theory has been applied to study brain connectivity, as well as other biological networks, including cellular metabolism, gene regulation, and ecology (Bullmore and Sporns, 2009; Rubinov and Sporns, 2010; Nandagopal and Elowitz 2011; Alexander 2013; Rajan et al. 2015; Jafarzadeh and Iranmanesh 2016a, b; Bordier et al. 2017; Mijalkov et al. 2017; Erciyes 2023).

An important area of brain connectivity research is the study of the functional connectivity of multiple spike trains. Functional connectivity refers to the statistical dependencies and influences between spike trains. After applying statistical techniques to the multiple spike trains, a binary, directed connectivity matrix is obtained, where a non-zero entry indicates a directed connection between two neurons. This connectivity matrix defines a graph, whose structural properties can be quantified further (Gerhard et al. 2011). Functional connectivity can be estimated in various ways, such as pairwise cross-correlation analysis (Perkel et al. 1967; Aertsen et al. 1989) and statistical models (Brown et al. 2004; Truccolo et al. 2005; Schneidman et al. 2006; Pillow et al. 2008; Paninski et al. 2010; Masud and Borisyuk, 2011; Chen et al. 2024; Guha et al. 2024). The basis to all functional connectivity analyses is time series data from neural recordings, extracted from functional magnetic resonance imaging (fMRI), electroencephalography (EEG), magnetoencephalography (MEG), and multielectrode arrays (MEA) (Salvador et al. 2005; Wu et al. 2007; Fiecas et al. 2010; Kostelecki et al. 2011; Hadley et al. 2016; Mele et al. 2019; Bruzzone et al. 2022).

Functional connectivity analysis is a vital tool in neuroscience, with stimulus type playing a crucial role in modulating neural networks. Traditional functional connectivity studies often used resting-state or controlled task-based paradigms, but recent advances in neuroimaging have enabled the use of more ecologically valid stimuli, such as movies, music, and narratives, which engage multiple cognitive processes and enhance intersubject correlation (Hasson et al. 2008; Sonkusare et al. 2019). Despite their advantages, naturalistic stimuli pose challenges in separating stimulus-driven activity from intrinsic fluctuations. Controlled tasks, like adaptive working memory paradigms, remain valuable for isolating specific cognitive functions and examining dynamic functional connectivity shifts under varying cognitive demands (Cohen et al. 2008). Multimodal stimuli further broaden the scope by enabling cross-modal functional connectivity investigations (Kayser et al. 2009). Additionally, emerging methods such as closed-loop neurofeedback and real-time fMRI allow researchers to causally probe and modulate functional connectivity, offering promising therapeutic avenues for psychiatric conditions (Ros et al. 2013; Sitaram et al. 2017).

Resting-state functional connectivity reveals intrinsic network architecture while stimulus-driven functional connectivity captures how external inputs dynamically reconfigure these networks to support perception, cognition, and behavior (Hutchison et al. 2013). Understanding the biological mechanisms underlying stimulus-evoked functional connectivity is essential for bridging brain dynamics with cognitive processes and clinical applications (Bassett and Sporns 2017).

Different stimuli—from sensory inputs to cognitive tasks—engage distinct neurobiological processes, such as thalamocortical loops for sensory processing or frontoparietal networks for working memory, mediated by mechanisms like glutamatergic signaling and dopaminergic modulation (Saalmann et al. 2012; Cohen and D’Esposito 2016). Clinically, aberrant functional connectivity responses are linked to disorders like anxiety and Alzheimer’s, where disrupted network dynamics correlate with symptoms and pathology (Buckner et al. 2009; Etkin et al. 2015). Advances in multimodal imaging, such as fMRI-MRS and optogenetics, elucidate cellular and molecular substrates of functional connectivity, while naturalistic paradigms provide ecologically valid insights into brain function (Hasson et al. 2004; Lee et al. 2010; Mullinger et al. 2017).

### Introduction: Graph theory

Core measures of graph theory include segregation, which refers to the degree to which network elements form separate clusters and is associated with the clustering coefficient (Rubinov and Sporns 2010). Integration refers to the network's capacity to become interconnected and exchange information, defined by the network’s characteristic path length (Rubinov and Sporns 2010). Other measures include density, node degree, graph efficiency, betweenness centrality, and motif analysis (Milo et al. 2002; Sporns and Kotter 2004; Rubinov and Sporns 2010). Application of graph theory is widespread. For example, the P1 model (Holland and Leinhardt 1981) is used in social science networks to identify influential and attractive individuals within a network.

Probably, the first attempt to apply graph theoretical concepts to fMRI was a methodological paper by Dodel et al. (2002) and the first application of graph theory to MEG data was published in 2004 (Stam 2004), and to EEG data in 2007 (Stam et al. 2007). Over the past ten years, connectivity between different brain areas has been intensively studied using data from fMRI, EEG, or MEG (Vico Fallani et al. 2014; Liu et al. 2017; Islam et al. 2017; Vecchio et al. 2017; Shamshiri et al. 2019; Warbrick 2022; Chiarion et al. 2023; Fang et al. 2023; Tanamachi et al. 2023). In addition to these studies, there have been studies focused on MEA signals (Gerhard et al. 2011; de Abril et al. 2018). Most studies estimated the connection matrix based on pairwise measures and used undirected measures. These pairwise measures typically focus on pairs of spike trains but fail to account for all possible influences from other simultaneously recorded spike trains, which can lead to inaccuracies. Therefore, new techniques are required to capture all possible influences and accurately estimate functional connectivity.

### Introduction: Experimental data and a graph of functional connections

To find the functional connectivity (connections’ graph) we use the Cox method (Masud et al. 2011). This method captures all possible influences between simultaneously recorded spike trains and allows to estimate an accurate functional connectivity. Here we investigate the functional connectivity using the graph theory measures. We apply the graph theory approach to graphs of functional connections of experimental recordings from the visual cortex of a cat under different stimuli. In fact, our results relate to two projects: 1) Experimental study of primary visual cortex of anesthetized cat under visual stimulation by moving bars of particular orientation; 2) Computational study the simultaneously recorded spike trains from project 1 to find the functional connectivity graph, calculate graph theory measures and find a correspondence between the functional connectivity and stimulation.

To investigate the functional connectivity of experimental data recorded from the visual cortex of a cat (see Appendix A, Nikolic 2007; Schneider et al. 2006), graph theory methods are applied to the connectivity matrix obtained using the Cox method. The experiment involves six different stimuli (various orientations of a moving grid), each repeated 20 times, resulting in 120 total applications of the stimuli. The order of stimulus presentation is randomized. During the experiment, the spiking activity of 32 channels is recorded.

For each stimulus, 20 intervals (each six seconds long) are selected to represent a total interval of 120 s for that stimulus. All spikes from these intervals are considered continuously, despite the gaps between intervals. This process results in 32 spike trains for each stimulus. For example, for stimulus 1, all subintervals corresponding to its application are selected, considered continuously, and all spikes are analysed for functional connectivity. This process is repeated for stimulus 2 and so on, creating six sets of 32 simultaneous spike trains. Each set corresponds to one stimulus. For each of the six stimuli, 32 spike trains are analysed to identify functional connectivity.

**Fig. 1:**
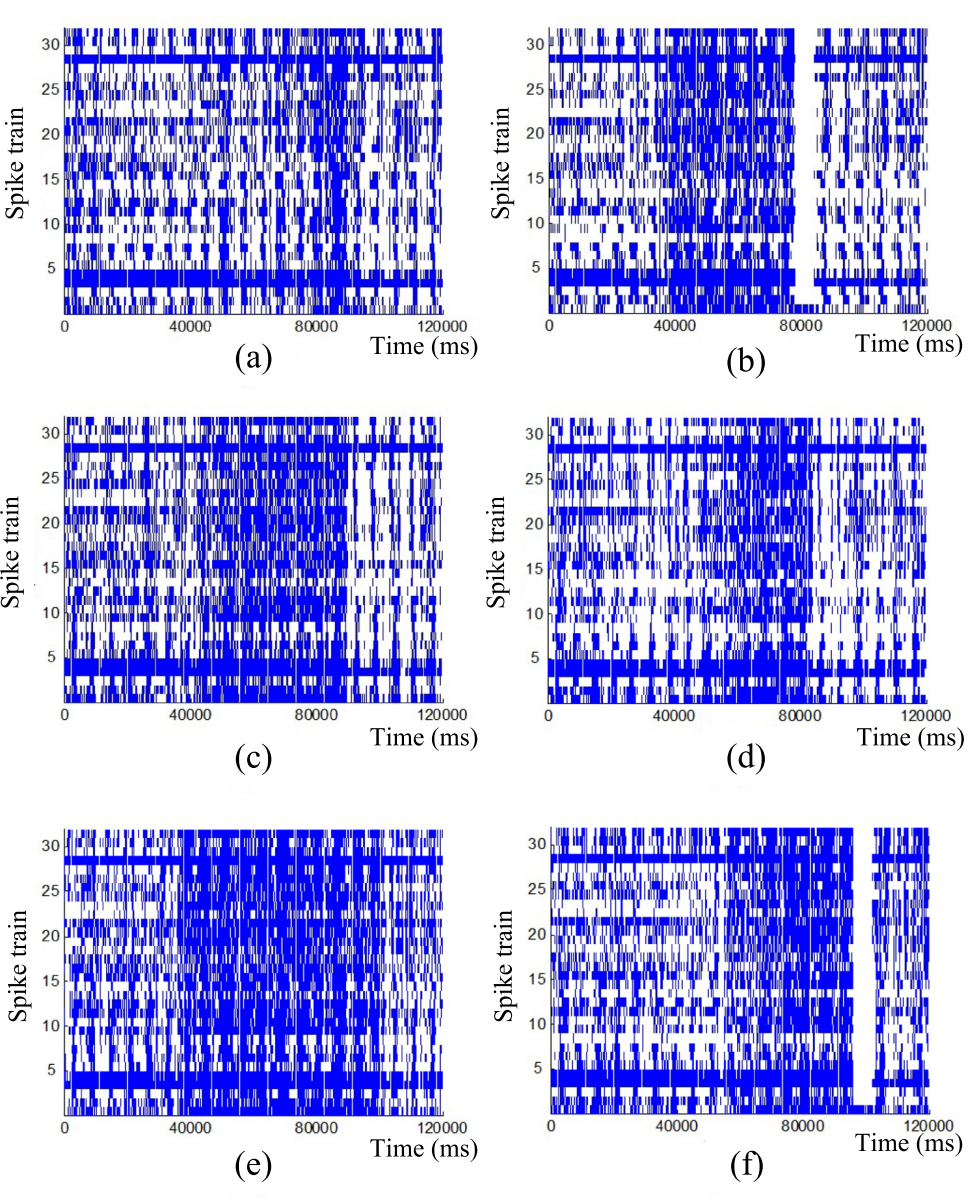
Raster plot of 32 spike trains across six stimuli. **a** Spike trains for Stimulus 1 **b** Spike trains for Stimulus 2 **c** Spike trains for Stimulus 3 **d** Spike trains for Stimulus 4 **e** Spike trains for Stimulus 5 **f** Spike trains for Stimulus 6

The raster plots of the 32 spike trains for six different stimuli are shown in Fig. 1 and Table 1 shows spiking rate for each channel (row) and each stimulus (column). It is known that for cortical neurons under typical experimental conditions, firing rates in the range of 5–20 Hz are often classified as medium (Buzsáki 2006). Analysis of the raster plots and firing rates reveals that three channels (#4, #5, and #29) exhibit significantly higher spiking rates than the others and for some stimuli they are outside of the interval for medium spiking rates. To ensure consistency in spiking rates among the spike trains, these three spike trains are considered to be the outliers and they are excluded from further analysis. The remaining 29 spike trains are used as they display similar spiking patterns. However, to avoid confusion we keep the original numbers of spike trains which have been prescribed the in experiment. Therefore, in our analysis we have no spike trains with numbers 4, 5, and 29.

**Table 1:** Firing rates of 32 spike trains in response to six different stimuli

Spike train	Firing rate					
	Stimulus					
	1	2	3	4	5	6
1	3	4.1	8.5	7.4	9	7
2	4.5	8	6.5	5.4	6.7	6.2
3	6.3	7.1	4.6	4.8	6.4	7.8
4	96.9	94.1	118.2	87.1	135.5	106.9
5	18.6	16.1	42.9	30.5	41.9	24.1
6	1.6	2	5.8	2.7	6	3.4
7	3	4	5.2	4.8	6.2	3.8
8	8	7.2	2.1	1.9	4.3	2.8
9	1	0.8	2.4	1.1	5.7	0.8
10	3.6	4.3	7.5	3.5	11.5	5.2
11	2.9	3.2	4	3.2	4.9	3.4
12	6.4	5.4	5.5	3.9	9.3	5.4
13	8.4	5.8	7.7	3.8	11	9.7
14	6.1	2	4	1.6	10.4	3.5
15	3.8	4.6	3.3	3.8	8.5	4.3
16	4.5	4.1	6.2	4.9	6.7	6.2
17	2.2	3.1	3.3	3.2	4.5	2.9
18	2.3	2.7	2.6	2.4	3.6	2.4
19	3.8	2.9	3.7	3.2	4.8	2.6
20	4.6	4.3	4.7	3.8	7.5	4.1
21	2.7	3.6	4.8	3.8	5.7	4.1
22	4.7	4.5	4.6	4.8	6.2	5.2
23	2.3	2.4	3.1	2.3	3.2	2.8
24	3.5	4.1	4.6	3.3	4.9	3.7
25	2.9	3.9	4.3	4.2	6.2	4
26	2.4	2.9	3.7	2.5	4.9	3.7
27	4.7	5.8	6.6	5.4	8.2	4.9
28	7.2	8.3	6	4.8	16.7	4.4
29	33.5	32.1	31.5	28.5	39.3	35.7
30	3.6	5.2	5	5.1	6.5	6.5
31	5.8	4.5	2.6	1.8	5.2	7
32	9.6	12.7	11.1	8.2	18	17.4

To summarise, the experimentally recoded set of spike trains includes six groups corresponding a particular stimulus (from 1 to 6). For each stimulus (from 1 to 6) the subgroup includes 29 spike trains numbered from 1 to 32 with exclusion of numbers 4, 5, and 29. For each stimulus, the subgroup of 29th spike trains has been simultaneously recorded during 120 s and these spike trains represent the spiking activity in the primary visual cortex in response to the particular stimulus.

Our goal is twofold: 1) for each stimulus to analyse simultaneously recorded spike trains and define the graph of functional connectivity; 2) for each stimulus apply graph theory methods to calculate the characteristic graph measures and study the functional connectivity which appears on presentation of the stimulus.

### Results: Connectivity matrix and diagram

**Fig. 2:**
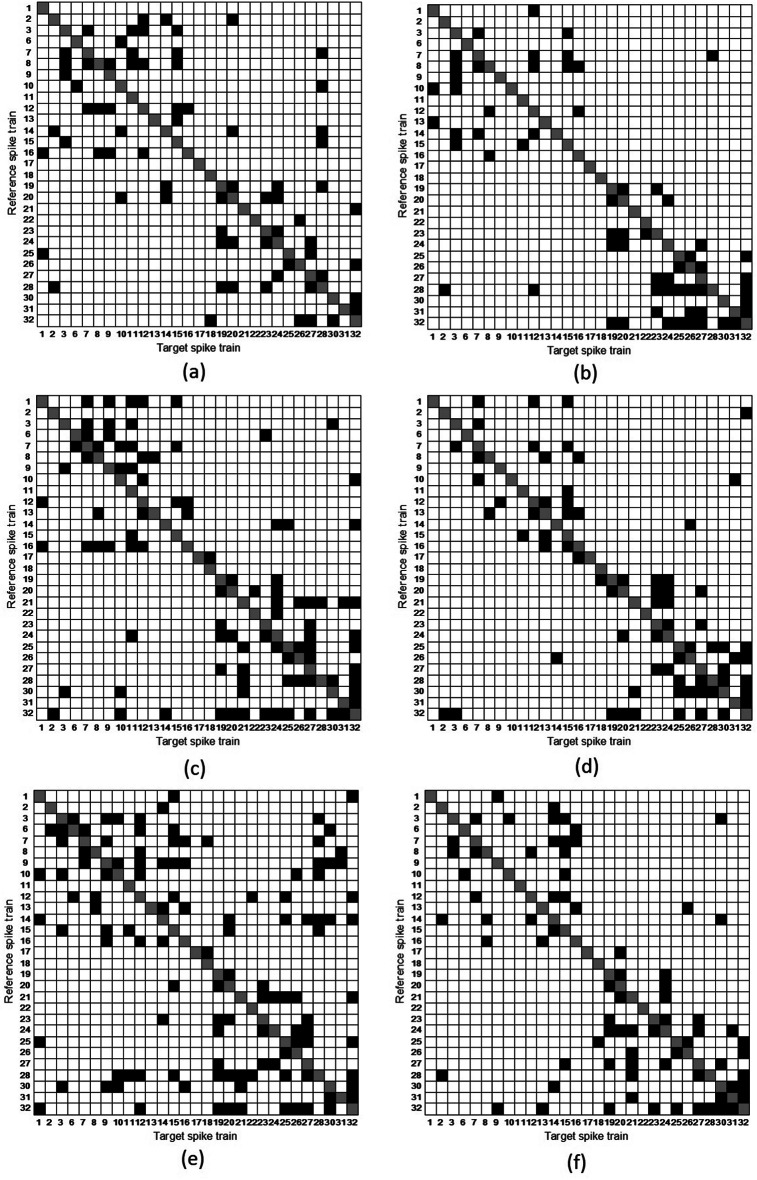
Connectivity matrices of 29 spike trains across six stimuli **a** matrix for Stimulus 1 **b** matrix for Stimulus 2 **c** matrix for Stimulus 3 **d** matrix for Stimulus 4 **e** matrix for Stimulus 5 **f** matrix for Stimulus 6. Note that the presence of a connection is represented by a black square, whilst the absence of a connection is represented by a white square. Main diagonals are indicated in grey and self-connections are excluded

To study the correspondence between stimuli and neuronal activity in the primary visual cortex, first, we find the functional connectivity between the spike trains for each stimulus. For finding the functional connectivity of 29 spike trains we use the Cox method (Masud and Borisyuk 2011). This method allows finding influences (connections) of spike trains to the selected target spike train. For example, for stimulus 1, we consider the spike tarin #1 as the target and find that spike trains #16 and #25 influence the target spike train #1. These influences (connections) are shown in the first column of the connectivity matrix at Fig. 2a. Also, these connections are shown by two arrows incoming to the node #1 (corresponding the spike tarin #1) in Fig. 9a.

Thus, for a selected spike train to be considered as the target spike train while the remaining 28 are treated as reference spike trains, we find all influences from the reference spike trains to the target spike trains. This process is repeated 29 times to obtain the complete functional connectivity of the 29 spike trains. The resulting functional connections, referred to as the connectivity matrix, are represented by squares in Fig. 2. The direction of functional connectivity is from the reference spike train to the target spike train (e.g. in the column #k of the connectivity matrix we show connections to the spike train #k).

Visual inspection of the connectivity matrix for each stimulus shows that connections tend to be grouped into two clusters: one cluster around the left-top corner and another cluster around the right-bottom corner of the matrix. The first cluster consists of the spike trains numbered #1–#16, while the second cluster includes the spike trains numbered #17–#32. This clustering occurs because the original 32 spike trains were recorded using two separate electrodes, each contributing 16 spike trains. It is interesting to note that for all stimuli there are no incoming connections to the spike train #7. Notably, spike trains #17 and #18 do not belong to either cluster.

To analyse this observation, we calculate several general characteristics of the connectivity graph: the density, the characteristic path length, the global efficiency, and the global clustering coefficient. The results of these calculations for each stimulus are shown in Table 2. Note: in Appendix we provide a short explanation of these and other graph characteristics.

**Table 2:** Four graph theory measures for each of the six stimuli

Stimulus	Density	Characteristic path length	Global efficiency	Global clustering coefficient
1	0.0874	3.1634	0.2456	0.2276
2	0.0764	2.7747	0.1871	0.2213
3	0.1170	2.8137	0.3428	0.3749
4	0.0874	3.1204	0.2278	0.2408
5	0.1429	2.3377	0.4041	0.2715
6	0.0936	3.0560	0.2939	0.2139

The connectivity matrices (Fig. 2) for the 29 spike trains across all six stimuli show low densities, ranging from 0.0764 to 0.1429 (Table 2) where Stimulus 5 has the highest density, indicating that for this stimulus connections are stronger compared to other stimuli. Additionally, note that Stimulus 1 and Stimulus 4 exhibit the same density.

The characteristic path length for all stimuli (Table 2) indicates that, on average, pairs of spike trains have long communication distances. Stimulus 5 exhibits the lowest characteristic path length, suggesting shorter communication distances between spike trains compared to the other stimuli. A related measure, global efficiency, which is the highest in stimulus 5 indicating the shortest communication distances among all six stimuli. The global clustering coefficient reveals that most spike trains across all stimuli do not form clusters with their neighboring spike trains, indicating weak connectivity. Stimulus 3 has the highest global clustering coefficient, suggesting some clustering among spike trains in this stimulus.

Stimulus 5, which exhibited the highest network density and global efficiency, suggests a highly interconnected network with shorter communication distances. Such a network structure might correspond to a high cognitive demand or heightened sensory processing under Stimulus 5. This finding may align with task-specific neural activation patterns observed in biological systems, where increased connectivity supports faster information transfer and integration.

The relatively high clustering coefficient in Stimulus 3 indicates some local modularity, potentially reflecting specialized processing within certain neural subgroups. The variations in these characteristics among stimuli highlight the dynamic adaptability of functional connectivity, which is crucial for task-specific cognitive operations such as attention, memory encoding, or sensory integration.

### Results: Clustering coefficients

**Fig. 3:**
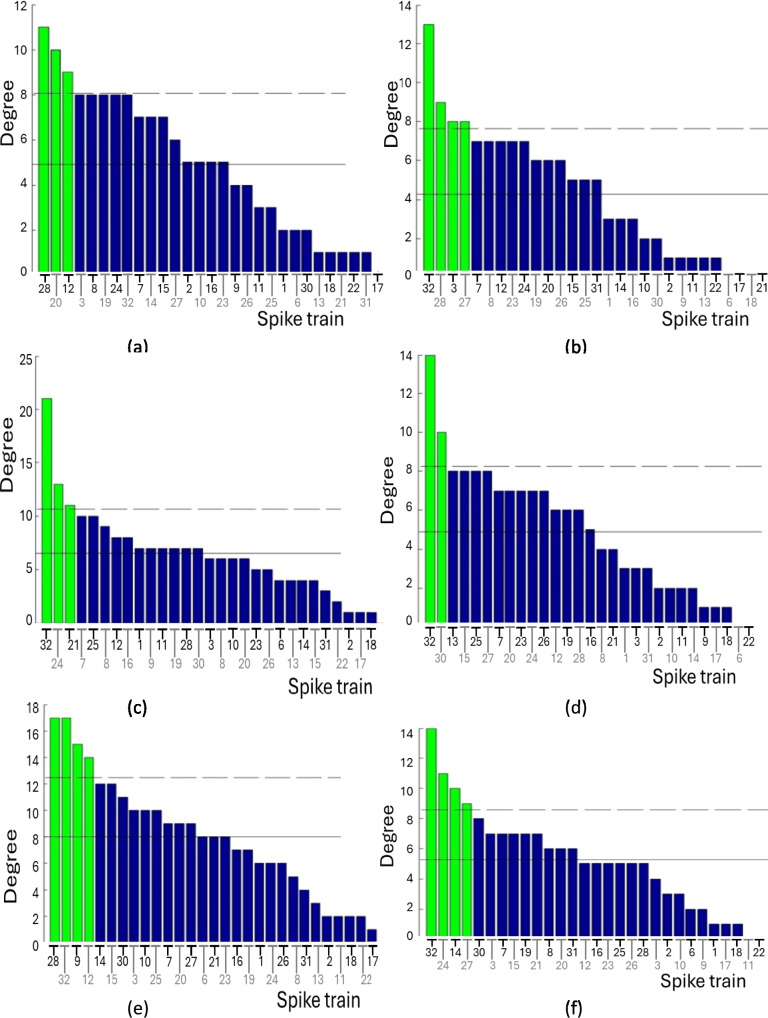
The degree of the spike trains is represented as descending bar charts for **a** Stimulus 1, **b** Stimulus 2, **c** Stimulus 3, **d** Stimulus 4, **e** Stimulus 5, and **f** Stimulus 6. The solid horizontal line indicates the mean degree of the spike trains, while the dashed horizontal line represents the mean plus one standard deviation. Bars exceeding one standard deviation are considered significant, with high-degree spike trains highlighted in green for clarity

Within graph theory the ‘outdegree’ of a vertex is defined as the number of outgoing edges from a vertex in a directed graph. The ‘indegree’ of a vertex is defined as the number of incoming edges incident on a vertex in a directed graph. The overall ‘degree’ is the sum of both the indegree and the outdegree. The degree of the spike trains, shown in Fig. 3 and Table 3, varies widely from 0 to 21. Some spike trains have very few connections (known as low-degree spike trains), while others have many (known as high-degree spike trains). High-degree spike trains are defined as having a degree greater than the mean plus one standard deviation of all spike trains (Sporns et al. 2007).

**Table 3:** Spike train number (#) and degree for each of the six stimuli

Stimulus	Spike train number(#) and degree
1	#28(11), #20(10), #12(9)
2	#32(13), #28(9), #3(8), #27(8)
3	#32(21), #24(13), #21(11)
4	#32(14), #30(10)
5	#32(17), #28(17), #9(15), #12(14)
6	#32(14), #24(11), #14(10), #27(9)

Spike train #32 has the highest degree in five of the six stimuli, except for stimulus 1. Spike train #28 has a high degree in three stimuli (1, 2, and 5), whilst spike trains #12, #24, and #27 have high degrees in two stimuli each. Spike trains #3, #9, #14, #20, #21, and #30 each have high degrees in only one stimulus.

The clustering coefficient is an important graph theory measure which indicates the density of connections among the neighbors of a spike train. Some spike trains have high clustering coefficients, indicating that their neighbors are also connected to each other. In contrast, some spike trains have low clustering coefficients, below the mean of all spike trains indicating that their neighbors are not connected to each other.

**Fig. 4:**
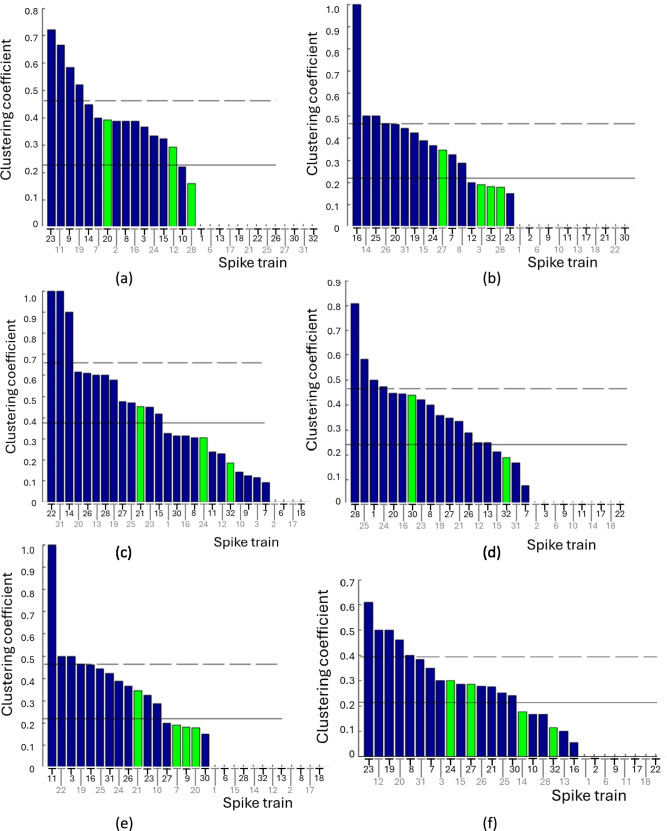
Clustering coefficients of the 29 spike trains displayed in bars of descending order for **a** Stimulus 1 **b** Stimulus 2 **c** Stimulus 3 **d** Stimulus 4 **e** Stimulus 5 **f** Stimulus 6. The solid horizontal line represents the mean clustering coefficient, while the dashed horizontal line represents the mean plus one standard deviation. Bars exceeding one standard deviation are considered significant, with high-degree spike trains highlighted in green for clarity

From Fig. 4 and Table 4, note that there are four spike trains (#16 for Stimulus 2, #22 and #31 for Stimulus 3, and #11 for Stimulus 5) that form complete clusters with their neighboring spike trains. Three spike trains #23 for Stimulus 1, #14 for Stimulus 3, and #28 for Stimulus 4 are strongly connected to their neighbors. Despite being influential and potential targets, the high-degree spike trains do not have strong connections with their neighbors (Table 5).

**Table 4:** Spike train number (#) and high and low clustering coefficient for each of the six stimuli

Stimulus	Spike train number(#) and clustering coefficient	
	High clustering coefficient	Low clustering coefficient
1	#23(0.722), #11(0.666), #9(0.583), #19(0.52)	#10(0.222), #28(0.16)
2	#16(1), #14(0.5), #25(0.5)	#12(0.2), #3(0.192), #32(0.182), #28(0.18), #23(0.15)
3	#22(1), #31(1), #14(0.9)	#10(0.142), #9(0.125), #3(0.115), #7(0.093)
4	#28(0.807), #25(0.583), #1(0.5), #24(0.473)	#15(0.221), #32(0.187), #31(0.166), #7(0.075)
5	#11(1), #22(0.5)	#28(0.197), #14(0.189), #32(0.179), #12(0.17), #13(0.166)
6	#23(0.611), #12(0.5), #19(0.5), #20(0.461), #8(0.4)	#32(0.114), #13(0.1), #16(0.055)

**Table 5:** Spike train number (#) and betweenness centrality for each of the six stimuli

Stimulus	Spike train number(#) and betweenness centrality
1	#28(0.291), #12(0.206), #2(0.188), #27(0.165)
2	#7(0.185), #28(0.174), #32(0.098), #8(0.096)
3	#32(0.415), #10(0.216)
4	#32(0.257), #7(0.162), #3(0.148), #30(0.148), #27(0.126)
5	#32(0.134), #14(0.131), #15(0.113), #12(0.109), #9(0.107), #28(0.105)
6	#32(0.277), #14(0.223), #30(0.223)

**Fig. 5:**
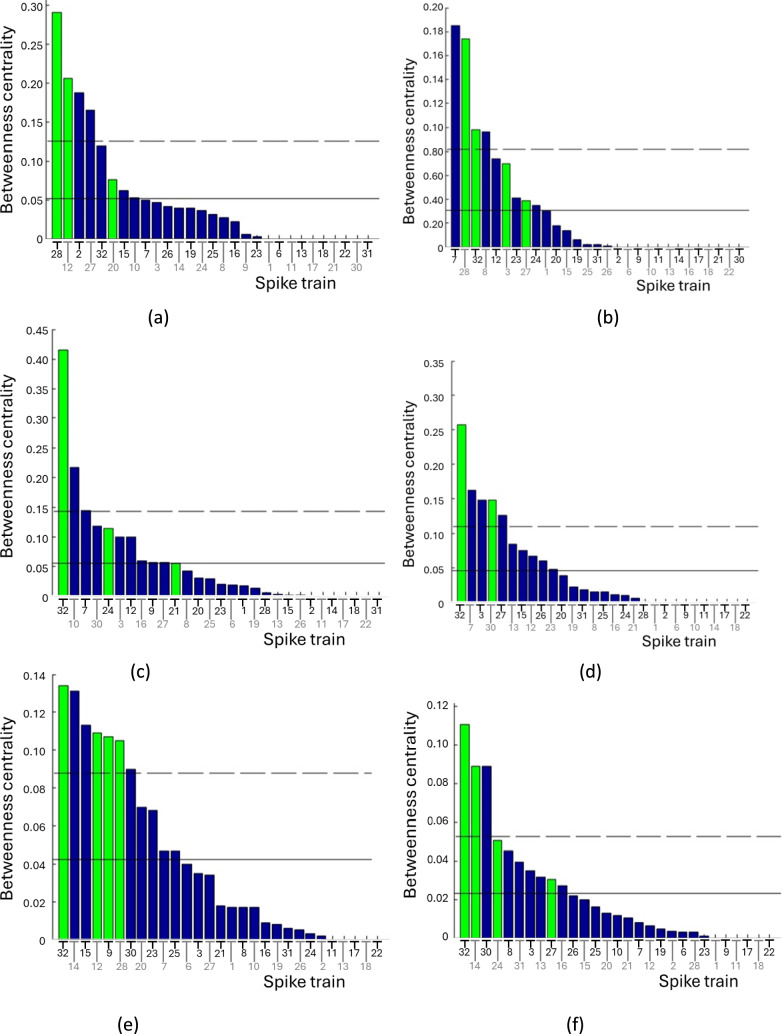
Betweenness centrality of the 29 spike trains, displayed in bars of descending order. The solid horizontal line represents the mean, and the dashed horizontal line represents the mean plus one standard deviation. Bars exceeding one standard deviation are considered significant, with high-degree spike trains highlighted in green for clarity. Panels show the betweenness centrality for each stimulus: **a** Stimulus 1 **b** Stimulus 2 **c** Stimulus 3 **d** Stimulus 4 **e** Stimulus 5 **f** Stimulus 6

Betweenness centrality of a spike train measures how much information passes through it. Overall, high-degree spike trains are central across all stimuli, transferring the most information. Spike train #32 is consistently central, except in stimuli 1 and 2 (Fig. 5).

### Results: Motif analysis

**Fig. 6:**
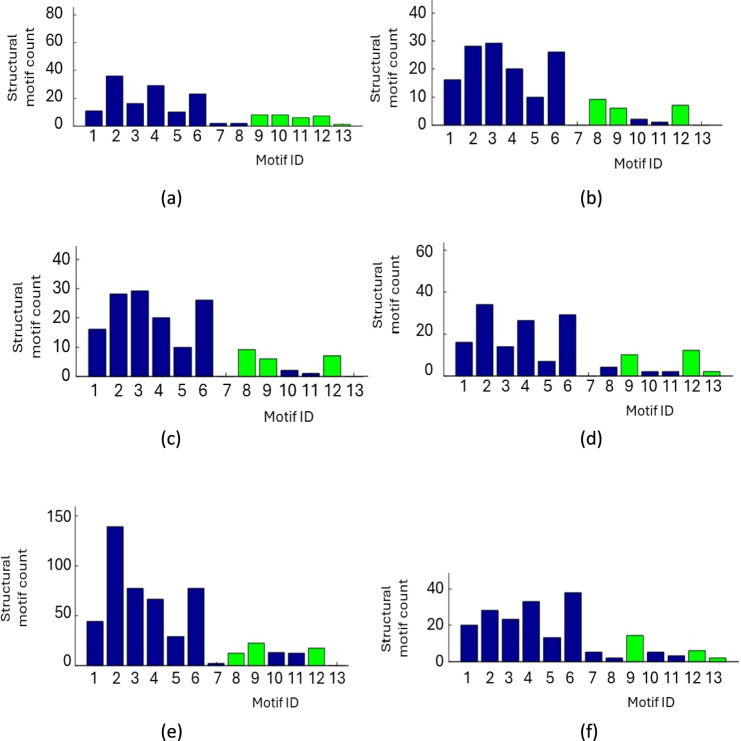
Structural motif count of size m = 3 among the 29 spike trains. Significant motifs are highlighted in green. Panels show motifs for each stimulus: **a** Stimulus 1 **b** Stimulus 2 **c** Stimulus 3 **d** Stimulus 4 **e** Stimulus 5 **f** Stimulus 6

To identify significant interconnections among the spike trains, motif analysis was conducted on the connection matrix derived from the six stimuli. Figure 6 and Table 6 illustrate the counts of structural motifs of size *m* = 3 within the 29-spike-train connection matrix across all stimuli. For *m* = 3, there are 13 distinct motifs, labelled ID-1 through ID-13. To determine statistically significant motifs, 1000 random networks were generated while preserving the in-degree and out-degree distributions of the spike trains. In Table 6, we observe that under Stimulus 1, motif ID-2 occurs 36 times—the highest count among all 13 motif ID’s. Other significant motifs include ID-9, ID-10, ID-11, ID-12, and ID-13. Analogous interpretation applies to the remaining stimuli.

**Table 6:** Motif ID with highest count and significant motif ID for each of the six stimuli

Stimulus	Motif ID	
	Highest motif and motif count	Significant motif
1	ID-2(36)	ID-9, ID-10, ID-11, ID-12, ID-13
2	ID-3(29)	ID-9, ID-10, ID-12
3	ID-6(62)	ID-8, ID-9, ID-12, ID-13
4	ID-2(34)	ID-9. ID-12, ID-13
5	ID-2(139)	ID-8, ID-9, ID-12
6	ID-6(38)	ID-9. ID-12, ID-13

Overall, Fig. 6 highlights Motif ID-2 and ID-6 as the most frequent across all stimuli. Motif ID-9 and ID-12 consistently emerge as significant motifs across the stimuli, indicating their role in the network structure.

The frequent emergence of motifs such as ID-2 and ID-6 and the significance of motifs ID-9 and ID-12 suggest a conserved structural organization across stimuli. These motifs could represent fundamental building blocks of neural computation, facilitating efficient information processing and robustness in the network. For instance, significant motifs might support repetitive firing patterns necessary for sustained attention or working memory.

### Results: Expansiveness and attractiveness coefficients

Another important measure in graph theory is the expansiveness and attractiveness coefficients (see description of P1 model in Appendix IX). A positive and large expansiveness coefficient of a spike train indicates a high probability that the spike train will influence other spike trains. A positive and large attractiveness coefficient indicates a high probability that the spike train is influenced by other spike trains. We summarise the highest values of both the expensiveness and attractiveness coefficients in Table 7.

**Table 7:** The highest values of expansiveness and attractiveness coefficients for each of the six stimuli

Stimulus	The highest expansiveness and attractiveness coefficients	
	High expansiveness coefficient	High attractiveness coefficient
1	#8(1.356), #16(0.904), #12(0.768), #20(0.619)	#15(1.022), #28(0.902), #27(0.687), #20(0.574)
2	#28(2.226), #32(1.739)	#3(1.366), #12(1.03), #27(0.839)
3	#32(1.761), #28(1.243), #16(1.116)	#27(1.435), #24(1.082), #19(1.002), #32(0.908)
4	#32(1.1484), #30(1.216)	#15(1.491), #23(1.175), #24(1.175)
5	#28(1.551), #9(1.163), #32(1.052)	#12(0.902), #32(0.733), #19(0.727), #15(0.725)
6	#32(1.735), #3(1.053)	#15(1.461), #14(1.052), #19(1.036), #21(1.036)

The number of spike train is indicated by a hash symbol (#) and the coefficient value is given in brackets, e.g. #8(1.356) shows that the spike tarin #8 has the highest value of expansiveness coefficient which is 1.356.

**Fig. 7:**
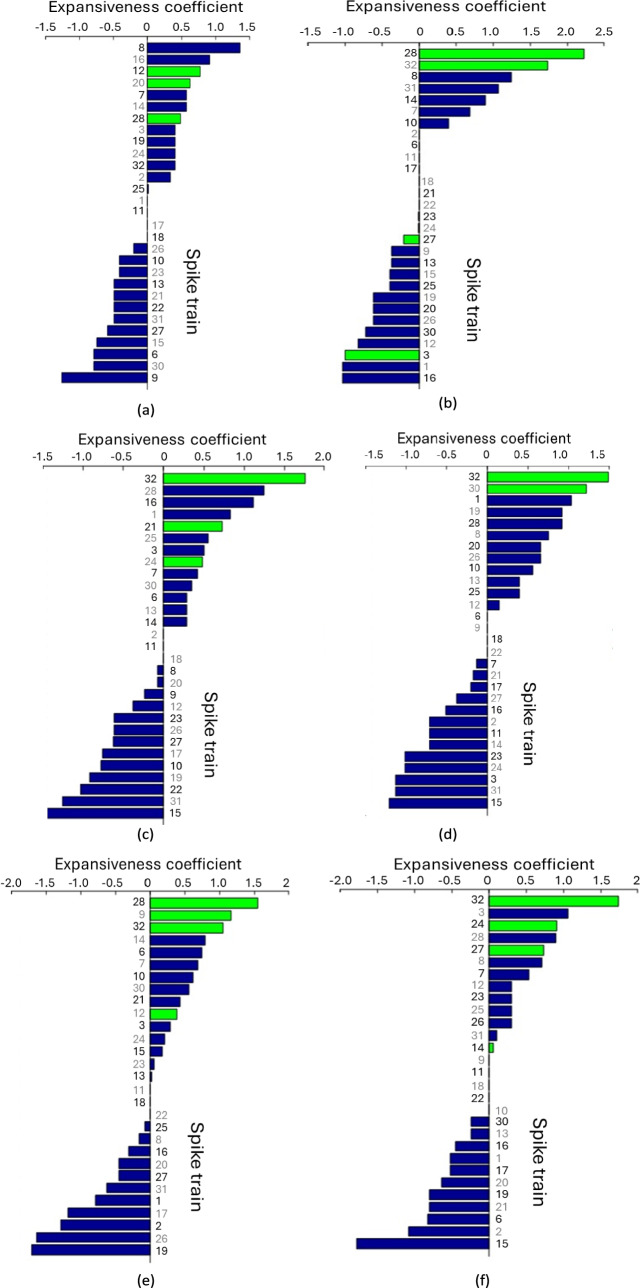
Expansiveness coefficient of the P1 model for 29 spike trains across six stimuli. **a** Stimulus 1 **b** Stimulus 2 **c** Stimulus 3 **d** Stimulus 4 **e** Stimulus 5 **f** Stimulus 6. Coefficients are displayed in bars of descending order. High-degree spike trains are highlighted in green

**Fig. 8:**
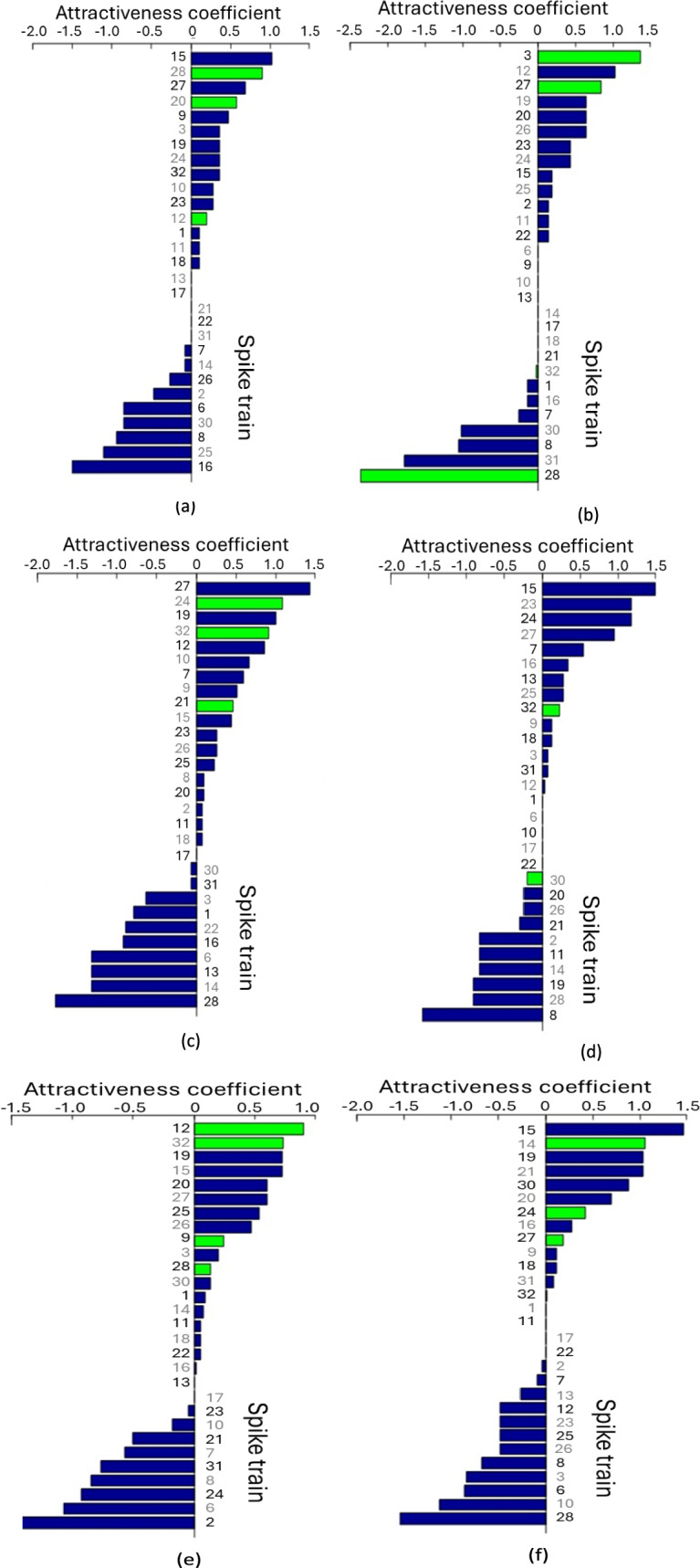
Attractiveness coefficient of the P1 model for 29 spike trains across six stimuli. **a** Stimulus 1 **b** Stimulus 2 **c** Stimulus 3 **d** Stimulus 4 **e** Stimulus 5 **f** Stimulus 6. Coefficients are displayed in bars of descending order. High-degree spike trains are highlighted in green

From the Figs. 7 and 8 and Table 7, it is evident that spike train #32 exhibits a high expansiveness coefficient in five stimuli and a high attractiveness coefficient in two stimuli. Spike train #28 shows a high expansiveness coefficient in stimuli 2 and 5, and a high attractiveness coefficient in Stimulus 1. Therefore, these spike trains are identified as the most influential and attractive in the dataset.

**Fig. 9:**
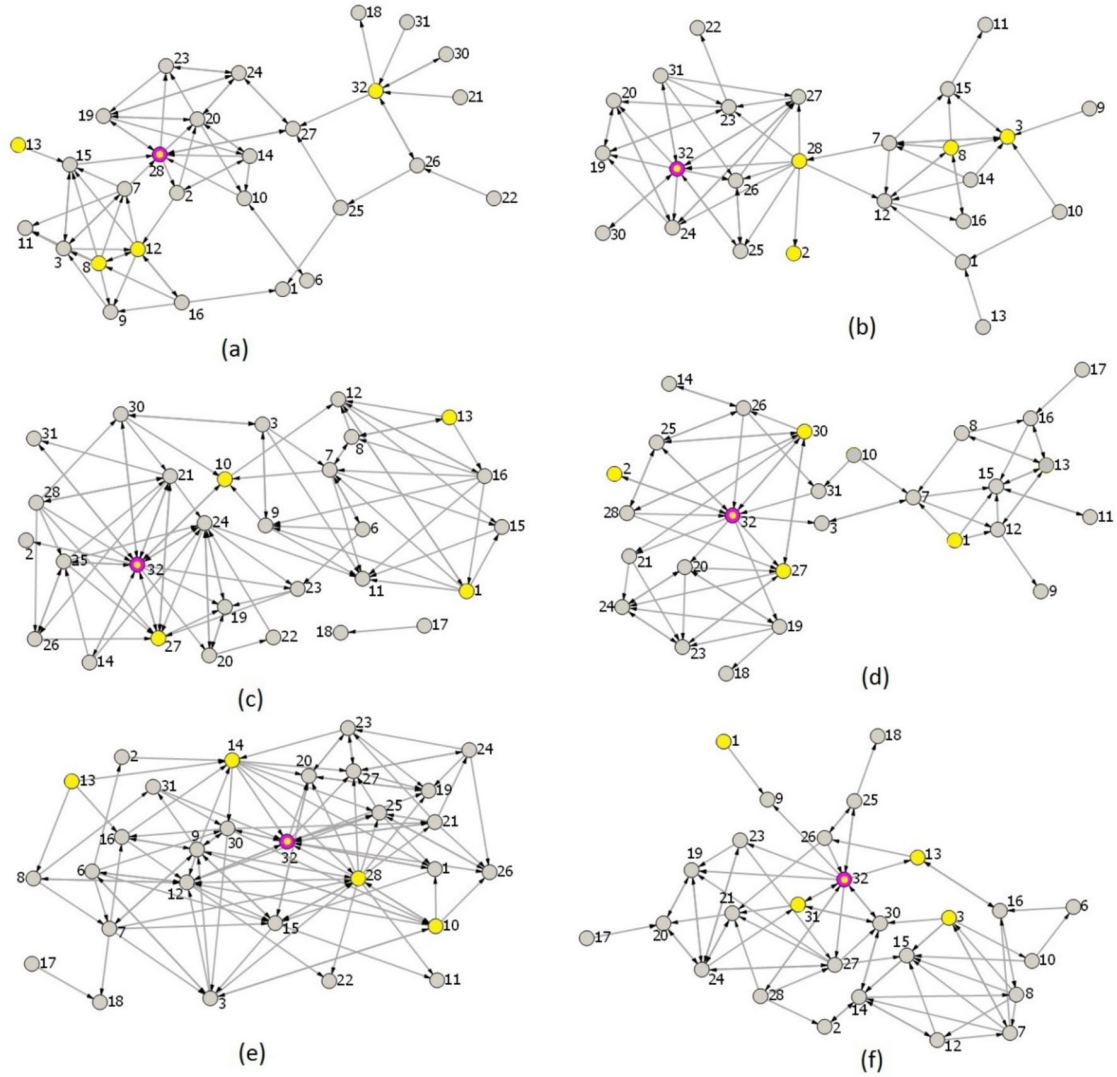
Diagrams (graphs) of functional connectivity showcasing the hub spike trains and the five most active spike trains for each of the six stimuli. **a–f** correspond to Stimuli 1–6, respectively. The hub spike train is represented by a magenta circle with a yellow centre, while the spike trains with the highest activity are highlighted in yellow

In graph theory, a critical concept is the hub, which denotes a spike train with substantially more connections than others in the network. These hub spike trains are pivotal to the network's structure and function, serving as central spike train through which significant information flows. Hub spike trains are typically identified based on their high degree or betweenness centrality (Sporns 2010). Analysis of degrees and betweenness centrality reveals the presence of hub spike trains across all stimuli. Figure 9 illustrates the hub spike train for each stimulus, with spike train #28 identified as the hub in Stimulus 1, and spike train #32 as the hub in all other stimuli. These spike trains, particularly #27, #28 and #32, play a crucial role in both transmitting and receiving information within the network of spike trains. In addition to hub spike train, Fig. 9 illustrates the five most active spike trains. Notably, these highly active trains generally exhibit high degree and betweenness centrality as well.

Notably, the connectivity diagrams for Stimulus 2 (Fig. 9b) and Stimulus 4 (Fig. 9d) share key characteristics: both display two clusters (left and right), with the left cluster being substantially larger in each case. This alignment in structure likely arises because Stimuli 2 and 4 each contain a vertical bar that moves—either to the right or to the left, respectively—suggesting a parallel in their underlying organization. Smaller right clusters have significant overlap of spike trains: #1, #7, #8, #9, #11–13, #15, and #16 are common for both right clusters. We hypothesise that the right cluster reflects the direction of bar movements: for Stimulus 2, spike trains #3 and #8 indicate moment to right; for Stimulus 4 spike train #1 indicates movements to left.

Stimuli 1–2 and 3–4 exhibit comparable bar movement directions, making them analogous in this respect. Analysing connectivity diagrams for these pairs, we observe that spike train #8 has high activity level and participates in both diagrams for Stimuli 1 and 2 (Fig. 9a, b) with high degree of connections but is not active enough in other connectivity diagrams. Probably, this spike train characterises the direction of bar movements to the right. Likewise, spike trains #27 has high activity level and participates in both diagrams for Stimuli 3 and 4 (Fig. 9c and (4)) with high degree of connections but this spike train is not active enough in other connectivity diagrams. Probably, this spike train #27 characterises the direction of bar movements to the left.

The identification of hub spike trains (#27, #28 and #32) as central elements of the network underscores their critical role in mediating information flow. These hubs may act as key integrators, analogous to highly connected neurons or brain regions like the praecuneus or hippocampus in biological systems, which are pivotal in cognitive processes such as attention or memory retrieval. The high activity and centrality of these spike trains suggest they could represent nodes of convergence where different stimuli evoke analogous responses, potentially reflecting shared neural pathways for processing related cognitive tasks.

**Fig. 10:**
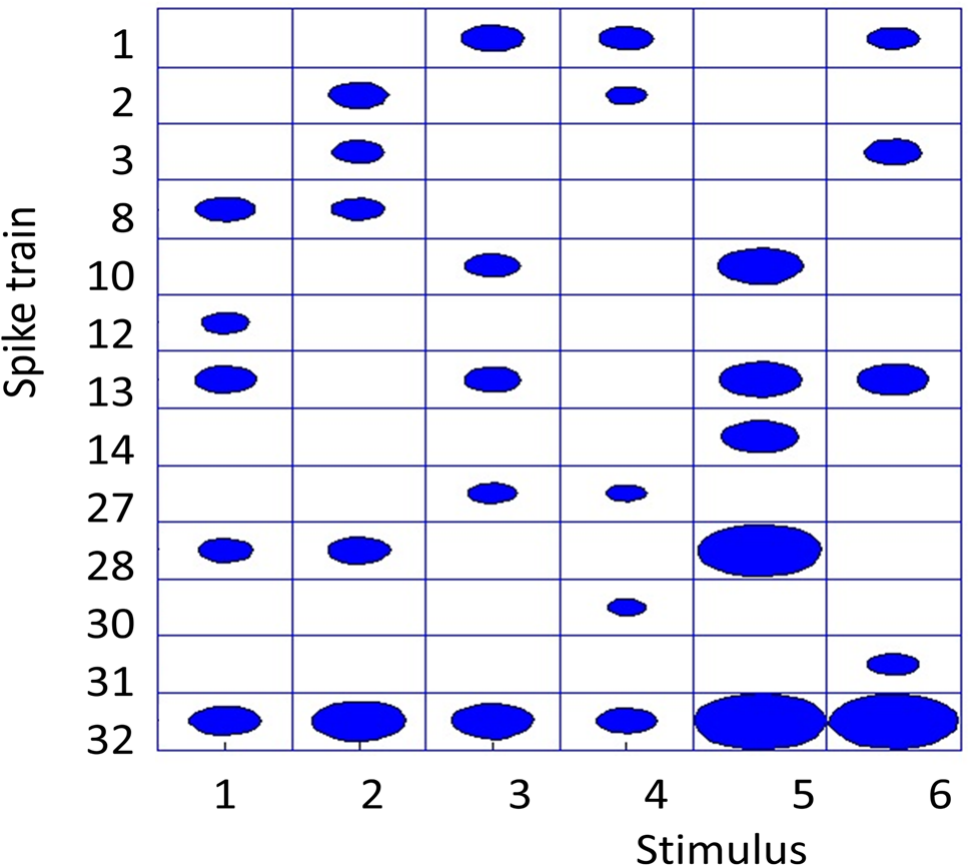
Top five most active spike trains for each stimulus. Circle size indicates spiking intensity

Figure 10 for each spike train indicates the highest level of spiking intensity for different stimuli. Spike train #32 exhibits the highest spiking activity across all stimuli, making it the hub spike train in different connectivity graphs. Spike train #13 shows the next highest activity levels in Stimuli 1, 3, 5, and 6. It is likely, that the neuron generating these spike trains for different stimuli specialises in finding of the orientation preference, moving bars of Stimuli 1, 3, 5, and 6 have the same orientation but different directions of movement. Likewise, the neuron generating spike trains #2 has the highest activity level for Stimuli 2 and 4 of the moving vertical bars in opposite directions.

The highest activity level of neuron generating spike trains #8 for stimuli 1 and 2 can reflect bar movements in the same direction for these stimuli. The highest activity level of neuron generating spike trains #27 for stimuli 3 and 4 can reflect bar movements in the same direction for these stimuli.

Spike trains #1 and #28 demonstrate high activity in Stimuli 3, 4, 6 and 1, 2, 5 respectively. The most active spike trains in Stimulus 5 (#10, #13, #14, #28, and #32) display the highest overall spiking intensity. Conversely, Stimulus 4 elicits the lowest spiking intensity.

To identify similarities in spiking activity patterns across the six stimuli, pair-wise correlation coefficients were calculated (Table 8). The analysis reveals that the spiking pattern of Stimulus 3 is highly correlated with that of Stimulus 4. This observation probably reflects that the two bars of stimulus 3 and the bar of stimulus 4 move in the same direction. Additionally, strong correlations exist between Stimuli 1 and 2 (again, two bars of stimulus 1 move at the same directions as the bar of stimulus 2).

**Table 8:** Pair-wise correlations between spiking activity of six stimuli

Stimulus	1	2	3	4	5	6
1	1	0.7889	0.4173	0.3222	0.6433	0.6715
2	0.7889	1	0.6079	0.6737	0.6542	0.7818
3	0.4173	0.6079	1	0.8213	0.741	0.78
4	0.3222	0.6737	0.8213	1	0.5405	0.7121
5	0.6433	0.6542	0.741	0.5405	1	0.6546
6	0.6715	0.7818	0.78	0.7121	0.6546	1

Pairwise correlations in spiking activity patterns between certain stimuli (e.g., Stimuli 3 and 4 or Stimuli 1 and 2) indicate the same direction of bar movement in stimuli. Also, these correlations could point to shared or overlapping neural processes. For example, Stimuli 3 and 4 may engage related sensory or cognitive pathways, such as those involved in visual or auditory discrimination tasks. These correlations might reflect stimulus-driven synchronization, a hallmark of coherent neural activity observed during focused attention or learning.

The network analysed in this study does not fully conform to the defining features of a small-world network. While some aspects, such as the presence of hubs and low density, align with small-world characteristics, the consistently low clustering coefficients and long characteristic path lengths are significant deviations. Stimulus 5, with its relatively lower path length and higher efficiency, may exhibit small-world-like properties more closely than the other stimuli. While the connectivity graph shares some properties with random networks (e.g., low density and clustering coefficients), other characteristics, such as the presence of hubs, significant motifs, and longer path lengths, indicate a deviation from randomness. The network demonstrates features of structured or biologically relevant systems rather than purely random connections. This suggests that the observed connectivity reflects functional or organizational constraints, supporting specific cognitive or sensory functions rather than stochastic processes.

## Discussion

Advancements in fMRI, EEG/MEG, and MEA technologies have facilitated the analysis of complex brain networks, enhancing our understanding of critical issues such as brain information processing modes and the mechanisms underlying cognitive functions. This study applies graph theory analysis to a network comprising 29 spike trains recorded from the visual cortex of a cat, revealing functional connectivity patterns derived using the Cox method.

The results obtained from graph theoretical methods exhibit analogous characteristics to previous studies. The average connection density across all connection matrices is 0.100, akin to findings in the macaque cortex study by Young (1993) where the density was 0.152. The average characteristic path length (2.877) closely mirrors that observed in the macaque cortex (2.312).

Analysis of individual spike train degree distributions reveals several spike trains with consistently high degrees across different stimuli. Remarkably, these high-degree spike trains exhibit low clustering coefficients, often below the mean of all spike trains. This phenomenon is consistent across each stimulus, aligning with previous findings (Sporns et al. 2007) which noted low clustering coefficients in high-degree areas of the macaque cortex and cat cortex.

Betweenness centrality analysis indicates that spike trains with high degrees also demonstrate high betweenness centrality, a trend observed consistently across different stimuli. This finding is consistent with Sporns et al. (2007), which highlighted high betweenness centrality in high-degree regions of the macaque cortex and cat cortex.

Examining the connection matrices of all stimuli, except Stimulus 1, reveals that spike train #32 consistently exhibits high degree and high betweenness centrality. This spike train emerges as a hub spike train, central in transmitting and receiving information among other spike trains. The existence of hubs in this type of data was also observed in studies by Yu et al. (2008) and Folias et al. (2013), where connections were assessed through the strength of gamma oscillations.

Further analysis of the matrices indicates that spike train #32 also displays a high expansiveness coefficient, a characteristic observed in five stimuli except Stimulus 1. Moreover, for stimuli 3 and 5, spike train #32 demonstrates a high attractiveness coefficient.

Across all stimuli, structural motifs ID-9 and ID-12 are consistently identified as significant. This finding parallels studies on the macaque cortex (Sporns and Kotter 2004; Sporns et al. 2007) which also highlighted the significance of structural motif ID-9 in network organization.

Current data clearly shows that the networks change as a function of stimulus properties. These changes are usually assumed to arise from the dynamics of interactions between neurons (Deco and Hugues 2012; Liang and Zhou 2022; Lobov et al. 2023). However, recently an alternative explanation has been proposed: transient re-wiring of neural networks by metabotropic receptors and G protein-gated ion channels (Nikolić 2023). Currently, it is not possible to decide which of the two possibilities is more likely in the present case.

The identified network properties, including the presence of hubs and significant motifs, suggest that the visual cortex dynamically reorganizes its connectivity to optimize processing based on the nature of the stimuli. For instance: the central role of spike train #32 in most stimuli implies that hubs may act as convergence points for integrating sensory inputs. Such structures are critical for higher-order cognitive functions like attention and sensory discrimination. The enhanced efficiency and reduced path lengths observed for Stimulus 5 suggest that the network prioritizes rapid information flow during specific sensory demands. This dynamic adaptability may underpin neural mechanisms for attention or decision-making.

This study excludes spike trains with exceptionally high firing rates, which could potentially affect the generalizability of the findings by omitting neurons with unique functional roles. Furthermore, the analysis is limited to the visual cortex, leaving it uncertain how the observed connectivity patterns and dynamics might generalize to other brain regions with different functional specializations.

## Conclusion

This study demonstrates the utility of graph theory in characterizing stimulus-driven changes in functional connectivity in the cat visual cortex. Key contributions include identifying stimulus-specific hubs, significant motifs, and their implications for cognitive activity. The findings align with previous studies while highlighting the dynamic adaptability of the visual cortex to sensory inputs. Future research should explore these dynamics in broader contexts, linking functional connectivity to cognitive performance and expanding to multi-regional analyses. These efforts will advance our understanding of how neural networks support adaptive behavior and inform both theoretical and practical applications in neuroscience and related fields.

## Figures and Tables

**Fig. 11:**
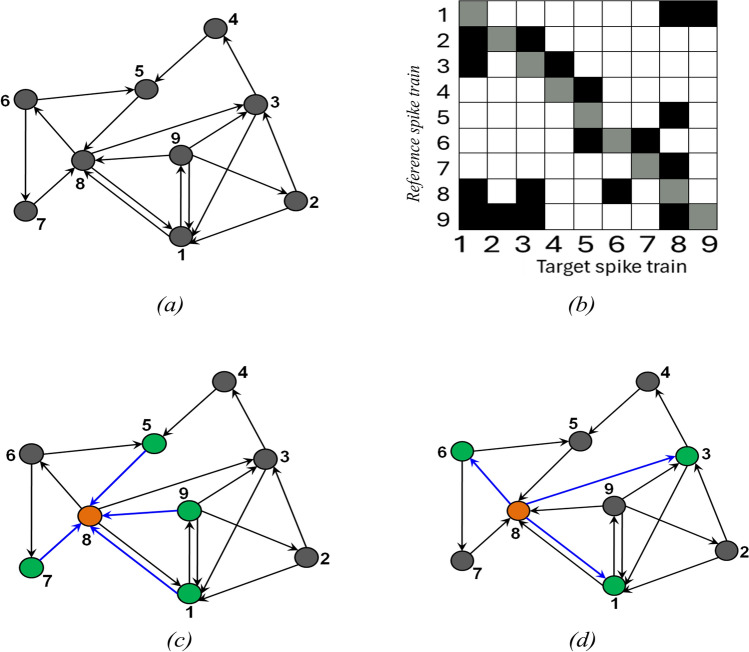
**a** Directed graph consisting of 9 nodes and 18 directed edges. The graph has 72 (9^2^–9) possible connections. The density of this directed graph is calculated as 18/72 = 0.25. **b** Adjacency matrix indicating the presence (black square) and absence (white square) of connections between nodes. The main diagonals are shaded in grey, and self-connections are excluded. **c** In-degree of node #8 (orange circle), which receives connections from nodes #1, #9, #5, and #7 (green circles). **d** Out-degree of node #8 (orange circle), which sends connections to nodes #1, #3, and #6 (green circles)

**Fig. 12:**
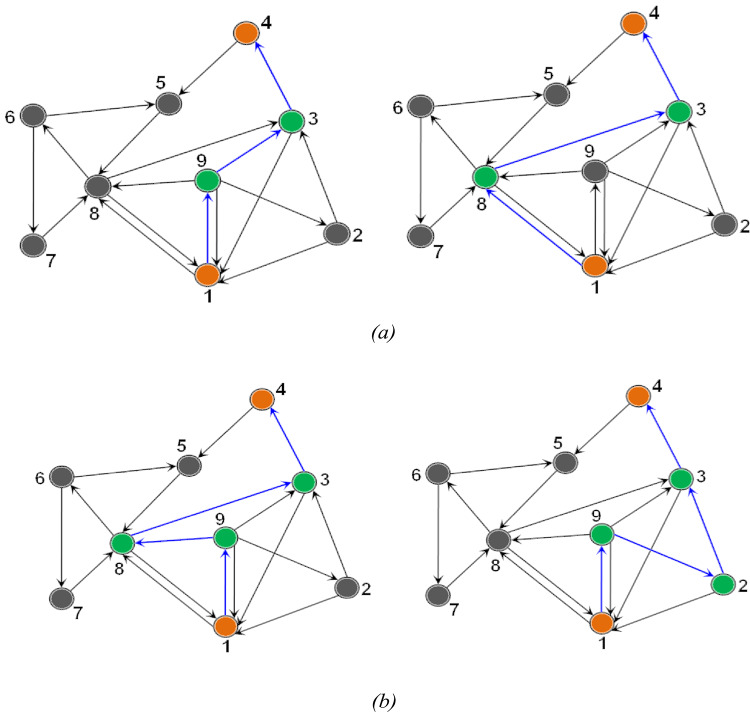
Denotes calculation of paths from node #1 to node #4. **a** Path from #1 to #4 of length 3, denoted by the sequence of nodes {#1, #9, #3, #4}, containing the directed edges (#1, #9), (#9, #3), and (#3, #4). An alternative path of the same length 3 is denoted by the sequence of nodes {#1, #8, #3, #4} (green circles). **b** Path from #1 to #4 of length 4, denoted by the sequence of nodes {#1, #9, #8, #3, #4} (green circles), containing the directed edges (blue) (#1, #9), (#9, #8), (#8, #3), and (#3, #4). An alternative path of the same length 4 is denoted by the sequence of nodes {#1, #9, #2, #3, #4} (green circles). The shortest possible path length from node #1 to #4 is 3, hence the distance from node #1 to node #4 is 3

**Fig. 13:**
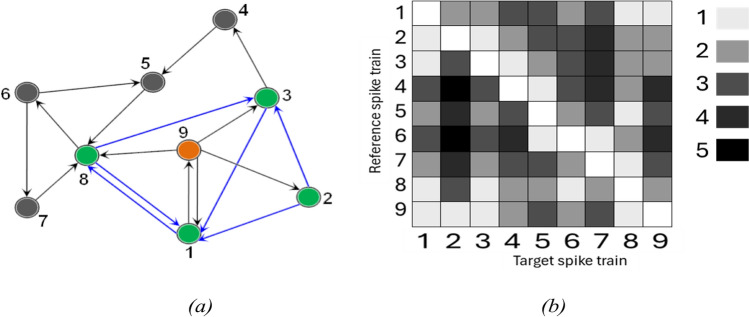
**a** Clustering coefficient of node #9 (orange circle). This node’s neighbours are #1, #2, #3 and #8 (green circle), which maintain 6 connections (blue edges) among them out of a possible 12 (4^2^–4). Therefore, the clustering coefficient of this node is 6/12 = 0.5. **b** Distance matrix of the 9-node graph, indicating the shortest path from node i (1,2 …, 9) to node j (1,2 … 9) i ≠ j. Pairwise distances are integers ranging from 1 to a maximum of 5

**Fig. 14:**
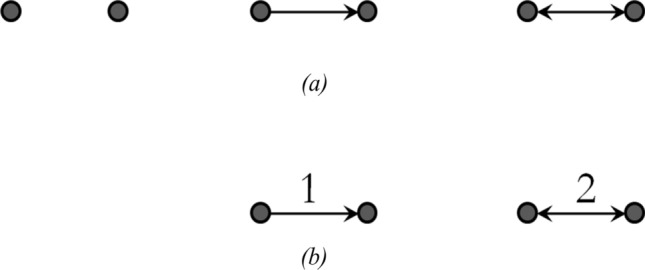
**a** The 3 directed graph of 2 vertices. **b** The 2 motif ID of 2 vertices

**Fig. 15:**
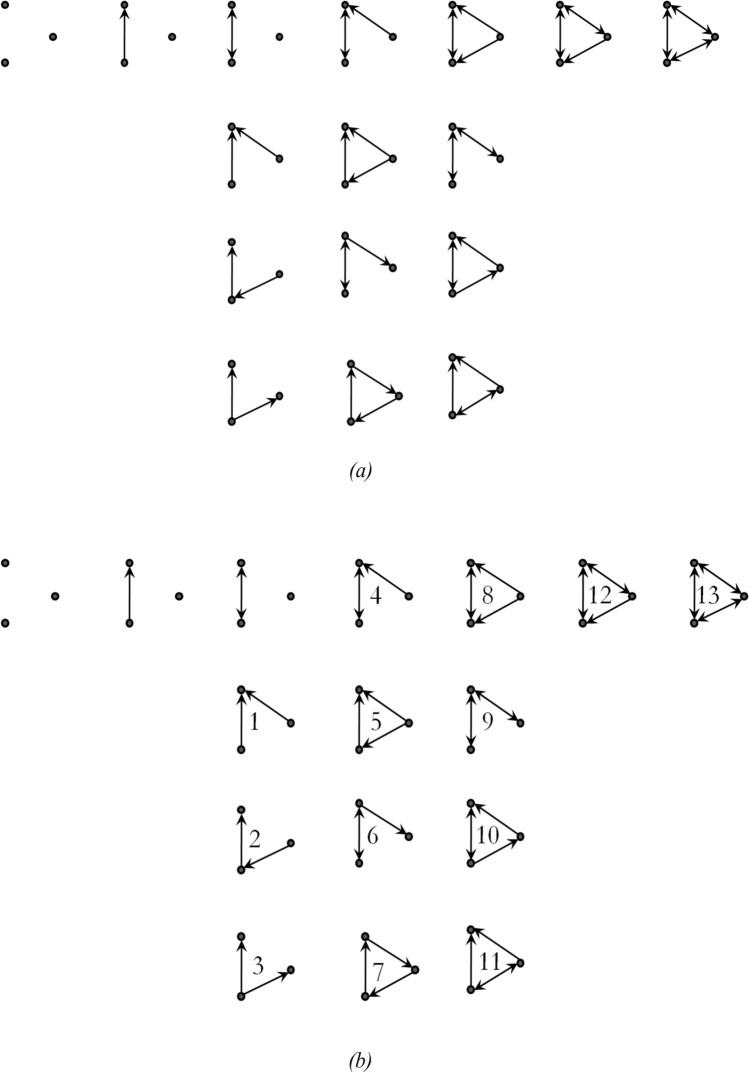
**a** The 16 directed graph of 3 vertices. **b** The 13 motif ID of 3 vertices

## Appendix A: Methods for data acquisition

### Preparation and recordings

Anesthesia was induced with ketamine and after tracheotomy, was maintained with a mixture of 70% N_2_O and 30% O_2_, and with halothane (0.4–0.6%). The cats were paralyzed with pancuronium bromide applied intravenously (Pancuronium, Organon, 0.15 mg kg^−1^ h^−1^). Multiunit activity (MUA) was recorded by using a silicon-substrate microelectrode arrays implanted to the cat’s primary visual cortex. The array or 16-channel probe (organized in a 4 × 4 spatial matrix) was supplied by the Center for Neural Communication Technology at the University of Michigan (Michigan probes). The probe had intercontact distances of 200 *μ*m. Signals were amplified 1000 times, filtered between 500 Hz and 3.5 kHz, and digitized with 32 kHz sampling frequency. The probe was inserted into the cortex approximately perpendicular to the surface, which allowed us to record simultaneously from neurons at different depths and along an axis tangential to the cortical surface.

### Visual stimulation

Stimuli were presented on a 21 inch computer monitor (HITACHI CM813ET, 100 Hz refresh rate). The software for visual stimulation was a commercially available stimulation tool, ActiveSTIM (www.ActiveSTIM.com). Binocular fusion of the two eyes was achieved by mapping the borders of the respective receptive fields (RFs) and then aligning the optical axes with an adjustable prism placed in front of one eye.

The stimuli consisted of either one white bar or two bars moving with the same speed in different directions (60-degree difference in orientation of two bars). In the stimuli with two bars, the bars crossed their paths at the center of the RF cluster. At each trial, the stimulus was presented in total for 5 s, but only 2 s with the strongest rate responses were used for the analysis. The bars appeared at about 3 degrees eccentricity of the center of the RF cluster and moved with a speed of 1 degree per second, covering the cluster of RFs completely.

In the six stimulation conditions the bars moved in the following directions: 1: 30 and 330 degrees; 2: 0 degrees; 3: 150 and 210 degrees; 4: 180 degrees; 5: 30 and 150 degrees; 6: 210 and 330 degrees. Each stimulation condition was presented 20 times, and the order of conditions was randomized across trials.

**Table:**

Stimulus	Direction	Stimulus	Direction
1		4	
2		5	
3		6	

## Appendix B: Graph theory methods

A graph serves as a mathematical model representing a system made up of interconnected elements, characterized by nodes and edges. Nodes symbolize fundamental components of the system, such as neurons within a region of the brain, while edges denote connections between pairs of nodes. In this context, edges are directed, representing connections from one node to another. The discussion herein focuses on graph theoretical methods applied to directed graphs.

In graph theory terminology, a directed graph *G*_*nl*_ consists of *n* nodes and *l* directed edges, where *l* ranges from 0 (for a null graph) to *n*^*2*^* – n* (for a fully connected graph excluding self-connections). The adjacency matrix, *A(G),* of the graph is composed of binary entries *a*_*ij*_, where *a*_*ij*_ = 1 indicates a connection from node *i* to node *j*, and *a*_*ij*_ = 0 indicates no connection. The diagonal elements *a*_*ij*_ of the adjacency matrix are zero to exclude self-connections, and *a*_*ij*_ does not necessarily equal *a*_*ji*_.

### Evaluating the density

The density *k*_*den*_ of an adjacency matrix *A(G)* is calculated as the number of its non-zero entries divided by the maximum possible number of connections. This measure ranges from 0 to 1, where 0 indicates a null graph (no connections), and 1 indicates a fully connected graph (all possible connections exist). In neural networks, the highest connection densities are typically observed among cortical areas and the pathways linking them. For matrices describing connection pathways between cortical areas, densities often fall within the range of *k*_*den*_ ~ 0.2–0.4 (Sporns 2002).

### Evaluating the degree

The adjacency matrix enables calculation of another fundamental graph measure, the degree. In a directed graph, the indegree and outdegree correspond to the number of incoming and outgoing edges, respectively (Fig. 11c, d). A node with a high indegree is influenced by many other nodes, whereas a node with a high outdegree has many potential functional targets. The indegree and outdegree of a node $i$ can be calculated as

$$\begin{array}{c}{k}_{i}^{in}=\sum_{j\in N}{a}_{ji}\\ {k}_{i}^{out}=\sum_{j\in N}{a}_{ij}\end{array}$$

where *N* is the set of all nodes in the network.

### Characteristic path length

Nodes can be connected directly by single edges or indirectly through sequences of intermediate nodes and edges. Ordered sequences of unique edges and intermediate nodes are known as paths (Fig. 12). If a finite path exists between two nodes, then one node can be reached by traversing a sequence of edges starting from the other node. In a directed graph, the length of a path equals the number of edges it contains. Paths of varying lengths represent possible routes for signals to travel indirectly between nodes. Longer paths typically have less impact than shorter ones. Most analyses focus on the shortest paths (distances) between nodes, as these paths are likely the most efficient for inter-node communication. The directed distance from node *i* to node *j*, (*d*_*ij*_), represents the length (number of edges) of one of the shortest paths from node *i* to node *j*.

The distance between two nodes is often of particular interest. The structure of the adjacency and distance matrices (Fig. 12b) collectively describes the pattern of communication within the graph's nodes.

One of the most commonly used measures in brain networks is the characteristic path length. This is computed as the global average of the graph's distance matrix (Watts and Strogatz 1998). The characteristic path length is calculated as

$$L=\frac{1}{n}\sum_{i\epsilon N}\frac{\sum_{j\epsilon N, j\ne i}{d}_{ij}}{n-1}$$

The characteristic path length is a global characteristic that measures how integrated a graph is and how efficiently information can be transmitted across the network. Shorter path lengths are typically more effective in facilitating information transfer between nodes. Therefore, the average path length of a network serves as an indicator of its capacity for global information exchange.

### Efficiency

A measure related to characteristic path length, and often more robust, is global efficiency (Latora and Marchiori 2001). It is computed as the average of the inverse of the distance matrix. A fully connected graph has maximal global efficiency since all distances are equal to one (every pair of nodes is directly connected by an edge). Conversely, a fully disconnected graph has minimal global efficiency because all distances between nodes are infinite. High efficiency indicates that pairs of nodes, on average, have short communication distances and can be reached in a few steps. The efficiency of a graph should be compared to that of a random network with the same indegree and outdegree for the nodes. The global efficiency is calculated as:

$$E=\frac{1}{n}\sum_{i\in N}\frac{\sum_{j\in N, j\ne i}{\left({d}_{ij}\right)}^{-1}}{n-1}$$

### Clustering coefficient

The clustering coefficient is a measure of the degree to which nodes in a graph tend to cluster together. It is one of the most elementary measures of local segregation within a network (Watts and Strogatz 1998). There are two versions of this measure (i) the local and (ii) the global clustering coefficients.

The local clustering coefficient of an individual node measures the density of connections among its neighbors. Neighbors are those nodes that are connected, either through an incoming or outgoing connection, to the node (Fig. 13a). Densely interconnected neighbors form a cluster around the node, while sparsely interconnected neighbors do not. The clustering of a node is high if its neighbors are also neighbors of each other.

The average of the clustering coefficients for each individual node is known as the global clustering coefficient, representing the clustering of the entire graph.

The clustering coefficient *C*_*i*_ of a node *i* with indegree ${k}_{i}^{in}$ and outdegree ${k}_{i}^{out}$ is usually calculated as (Fagiolo 2007)

$${C}_{i}=\frac{\frac{1}{2}\sum_{\begin{array}{c}j,h\epsilon N\\ h\ne j, h\ne i,j\ne i\end{array}}\left({a}_{ij}+{a}_{ji}\right)\left({a}_{ih}+{a}_{hi}\right)\left({a}_{jh}+{a}_{hj}\right)}{\left({k}_{i}^{out}+{k}_{i}^{in}\right)\left({k}_{i}^{out}+{k}_{i}^{in}-1\right)-2\sum_{j\epsilon N}{a}_{ij}{a}_{ji}}$$

The clustering coefficient *C*_*i*_ ranges between 0 and 1. Usually *C*_*i*_ is averaged over all vertices to obtain a mean *C* of the graph

$$C=\frac{1}{n}\sum_{i\epsilon N}{C}_{i}$$

### Betweenness centrality

Centrality measures in a graph help determine the relative importance of each node. These measures are often based on the concept of shortest paths. Betweenness centrality is particularly useful for understanding how much information flows through specific nodes in the graph. A node with high betweenness centrality can control the flow of information since it lies on the intersection of many shortest paths. The betweenness centrality of an individual node is defined as the fraction of all shortest paths in the network that pass through that node. The betweenness centrality of a node is calculated as follows (Freeman 1978):

$${C}_{i}^{B}=\frac{1}{\left(n-1\right)(n-2)}\sum_{\begin{array}{c}h,j\epsilon N\\ h\ne j, h\ne i,j\ne i\end{array}}\frac{{\rho }_{hj}(i)}{{\rho }_{hj}}$$

where *ρ*_hj_ is the number of shortest paths between *h* and *j*, and *ρ*_hj_*(i)* is the number of shortest paths between *h* and *j*, that pass through *i*.

### Motif analysis

Understanding the patterns of interconnections among multiple spike trains is crucial for elucidating their relationships. These interconnections are typically represented as a connected *m*-vertex graph, which is a subgraph of a larger graph. Motif analysis is used to identify these patterns of interconnections (Milo et al. 2002; Sporns and Kotter 2004). A motif is a connected subgraph of *m* vertices that occurs in a directed graph significantly more often than in randomized versions of the graph. These randomized versions have the same number of vertices, edges, and degree distribution as the original graph, but with edges distributed randomly.

A directed graph is a configuration whose figures are ordered pairs of points. In this context, the content of a figure is one or zero in respective accordance with the existence or non-existence of a directed line from the first member of the figure to its second member. Hence the figure counting series is 1 + *x*. Let us suppose *d*_m_*(x)* is the counting polynomial for directed graphs with *m* vertices. The counting polynomials *d*_m_*(x)* for *m* = 1 to 5 is provided by Harary and Palmer (1973):

$$ \begin{aligned} d_{1} \left( x \right) = & 1 \\ d_{2} \left( x \right) = & 1 + \, x\, + \, x^{2} \\ d_{3} \left( x \right) = & 1\, + \, x\, + \, 4x^{2} + \, 4x^{3} + \, 4x^{4} + \, x^{5} + \, x^{6} \\ d_{4} \left( x \right) = & 1\, + \, x\, + \, 5x^{2} + \, 13x^{3} + \, 27x^{4} + \, 38x^{5} + \, 48x^{6} \\ & + \, 38x^{7} + \, 27x^{8} + \, 13x^{9} + \, 5x^{{10}} + \, x^{{11}} + \, x^{{12}} \\ d_{5} \left( x \right) = & 1\, + \, x\, + \, 5x^{2} + \, 16x^{3} + \, 61x^{4} + \, 154x^{5} + \, 379x^{6} \\ & + \, 707x^{7} + \, 1155x^{8} + \, 1490x^{9} + \, 1670x^{{10}} \\ & + \, 1490x^{{11}} + \, 1155x^{{12}} + \, 707x^{{13}} + \, 379x^{{14}} \\ & + \, 154x^{{15}} + \, 61x^{{16}} + \, 16x^{{17}} + \, 5x^{{18}} + \, x^{{19}} + \, x^{{20}} \\ \end{aligned} $$

Using the counting polynomial *d*_*m*_*(x)* the numbers of directed graphs for *m* = 2 and *m* *= *3 vertices are shown in Figs. 14a and 15a. To obtain the number of motifs from the directed graph, all vertices must have either outdegree or indegree of at least one. Thus the number of motifs for *m* = 2 and *m* = 3 vertices are 2 and 13 respectively which are identified from Figs. 14a and 15a. The motif ID for *m* = 2 and *m* = 3 vertices are shown in Figs. 14b and 15b. For *m* = 4 and *m* = 5 the corresponding numbers of directed graphs are 218 and 9608; and the motif ID’s are 199 and 9364 (Harary and Palmer 1973). In this study motifs of size *m* = 3 are considered. There are some connected motifs that form a strongly connected graph. For *m* = 3 motifs with ID = 7, 9, 10, 12, and 13 are connected motifs. In a connected motif all vertices can be reached from all other vertices.

For multiple spike trains, the diagram of functional connectivity is identified using the Cox method, considering all spike trains simultaneously. From this connectivity diagram, the structural motif count can be determined by counting the number of distinct motif IDs. With the structural motif count for each distinct motif ID, a bar diagram of the structural motif count is created. Additionally, the diagram of functional connectivity can be obtained through the triplet analysis of the Cox method. This involves analyzing all possible triplets among the spike trains to identify a comprehensive diagram of functional connectivity.

To search the significant structural motifs from the diagram of functional connectivity, a large number of randomized diagrams (*n* = 100 or 1000) are generated. Note that the number of vertices and edges remain the same as the original diagram but the edges are distributed randomly. In order to quantify the significance of a given motif ID $i$, its Z-score can be computed (Boccaletti et al. 2006). If ${n}_{i}^{(real)}$ is the number of times that a motif ID *i* appears in the real diagram of functional connectivity, $<{n}_{i}^{(rand)}>$ and ${\sigma }_{i}^{(rand)}$ are the average and standard deviation of the motif ID *i* obtained from the randomized diagrams, then its Z-score can be computed as

$${Z}_{i}=\frac{{n}_{i}^{(real)}-<{n}_{i}^{(rand)}>}{{\sigma }_{i}^{(rand)}}$$

A structural motif is considered to be significant if the Z-score of this motif is higher than 2 (Sporns et al. 2007).

### The P1 model

The P1 model (Holland and Leinhardt 1981) of a graph identifies the relationships between nodes. For any pair of nodes in a graph, three possible relationships can exist (i) no ties (no edges in either direction between the nodes), (ii) an asymmetric tie; an edge going in one direction between the nodes but not the other, or (iii) a mutual tie; edges going in both directions between the nodes. These relationships are known as dyadic relationships and denoted by

$$ D_{{{\text{ij}}}} = \, ( \, A_{{{\text{ij}}}} , A_{{{\text{ji}}}} ), \, i \, \ne \, j $$

where *A* is the adjacency matrix.

A mutual relationship between node *i* and node *j* exists when *i → j* and *j → i* in the dyad which is denoted by *i ↔ j*. A mutual relationship is obvious when both the (*i,j)* and (*j,i)* cells are unity; that is *A*_ij_ = *1* and *A*_ji_ = 1, so that the dyad *D*_ij_ = *(1,1)*. The asymmetric dyad can occur in two ways, either *i → j* or *j → i* but not both. Specifically, *D*_ij_ = (1,0) or *D*_ij_ = (0,1). In null dyad the (*i,j)* and (*j,i)* symmetrically placed off-diagonal cells of *A* are both 0; that is, *A*_ij_ = *A*_ji_ = 1, implying that *D*_ij_ = (0,0). Thus, the three possible dyadic relationships can be represented as

$${D}_{ij}=\left\{\begin{array}{c}\left(\text{0,0}\right) \\ \left(\text{1,0}\right)\text{ or }\left(\text{0,1}\right) \\ \left(\text{1,1}\right)\end{array}\right.$$

A dyad with measurements on a directional relation consists of two nodes, *i* and *j*, and the possible ties between these two nodes. The ties between the nodes are viewed from the perspective of either node *i* or node *j*. From the perspective of *i* the relational variable *A*_ij_ records the possible ‘choice’ of* j* by *i*, while the relational variable *A*_ji_ records the possible ‘choice’ received by *i* from *j*. From the perspective of node *j* the relational variable *A*_ij_ records the possible choice of node *i* by node *j*, while the relational variable *A*_ij_ records the possible choice received by node *j*, from node *i*.

For a pair of nodes the ties in the dyad for both nodes is represented by a 2 × 2 array. There are two variables in this array. The first variable is indexed with *k*, which can be either 0 or 1, this codes the value of the tie sent by the row node *i* to the column node *j*. The second variable, also with two levels is indexed with *l*, this codes the value of the tie sent by the column node *j* to the row node *i*. So, the ties for each and every dyad can be presented in a 2 × 2 array. The new indices *k* and *l* are equal to either 0 or 1, depending on the state of the dyad.

Considering all dyads and the single dichotomous relation, there will be *g* × *g* binary matrix for *g* nodes. If each entry is replaced with the appropriate 2 × 2 table, a new contingency table is obtained of size *g* × *g* × 2 × 2. The first two dimensions of this table are indexed by the nodes. The size of the third and fourth dimensions is 2, and they are coded *k, l* = 0 or 1.

The *g* × *g* × 2 × 2 matrix is denoted by Y, and its entries are defined as follows:

$$ \begin{array}{*{20}c} {Y_{ijkl} = \, 1\quad if \, the \, dyad D_{ij} \, takes \, on \, the \, values \, (A_{ij} = k, \, A_{ji} = \, l)} \\ { = \, 0\quad otherwise.} \\ \end{array} . $$

The Y-array is a cross-classification of four variables. Thus, its entries have four subscripts. These are the nodes as senders *(i)*, the nodes as receivers *(j)*, and the relational variables *A*_*ij*_ (indexed by the third subscript, *k*) and finally *A*_*ji*_ (indexed by the fourth subscript, *l*). The (*i,j)* th cell of Y is not a single quantity, it is a 2 × 2 submatrix. In this 2 × 2 submatrix, there is a single 1 found in the (*k,l)*th cell. The remaining 2^2^ – 1 elements are 0. Thus, these submatrices are simply indicator matrices, giving the ‘state’ of each dyad.

To understand the Y matrix an example of two nodes is given. The matrix in Table 9 represents the ‘friendship’ of the nodes. The data shows that Node 2 does not name Node 1 as a ‘friend’ he likes, but Node 1 does nominate Node 2.

**Table 9:** Friendship of two nodes

Node	1	2
1	–	1
2	0	–

From Node 1’s perspective, the relational variable sent is *A*_*12*_ = *1*, implying that Node 1 likes Node 2 as a friend, and the relation received is *A*_*21*_ = *0*, implying that Node 1 is not liked as a friend by Node 2. From 2’s perspective, the relation sent is *A*_*21*_ = 0, Node 2 does not choose Node 1, and the relation received is *A*_*12*_ = *1*, Node 2 is chosen by Node 1. The recorded data for nodes 1 and 2 in this pair would be *D*_12_ = *(A*_12_*, A*_21_) = (1,0) so that *y*_1210_ = 1 while *y*_1200_ = *y*_1201_ = *y*_1211_ = 0. Similarly, *D*_21_ = *(A*_21_*, A*_12_*)* = (0,1) so that *y*_2101_ = 1 while *y*_2100_ = *y*_2101_ = *y*_2111_ = 0. The resultant Y array is shown in Table 10.

The P1 model is represented by a 4-dimensional Y-array. It focuses on the effects that signify the 'expansiveness' of nodes, the 'popularity' of their partners, and the 'reciprocation' of ties within dyads. The model primarily consists of three sets of parameters: one set describing nodes' tendencies to initiate ties (sending behavior), another describing nodes' tendencies to receive ties (receiving behavior), and a third describing interactions between pairs of nodes within dyads.

**Table 10:** Y matrix for the friendship of two nodes

	*J*					
*I*		*l* = *A*_ji_				
	*k* = *A*_ij_		0	1	0	1
		0	–	–	0	0
		1	–	–	1	0
		0	0	1	–	–
		1	0	0	–	–

The first set of parameters, known as expansiveness effects, reflects each node's inclination to nominate others as connections. The second set of parameters, popularity effects, indicates each node's likelihood of being nominated by others as a connection. Positive values of these parameters increase the probability of ties forming.

The third set includes parameters that represent reciprocity, measuring the extent to which dyads exhibit mutual ties rather than asymmetric ones. Positive reciprocity parameters enhance the likelihood of mutual ties between nodes.

The P1 model is expressed in four statements. Each of the four statements represents one of the four possible states of any given dyad: the null dyad *(A*_i,j_ = *A*_ji_ = 0, or* y*_*ij00*_ = 1), the mutual dyad, *(A*_i,j_ = *A*_ji_ = 1,* or y*_*ij11*_ = 1), and two cases of asymmetric dyads *(A*_i,j_ = 1,* A*_ji_ = 0, or* y*_*ij10*_ = 1, and* A*_i,j_ = 0,* A*_ji_ = 1, or* y*_*ij01*_ = 1).

In order to specify P1, the natural logarithm of the probabilities of each of these four dyadic states is represented as a function of several parameters:

$$logP\left({Y}_{ij00}=1\right)={\lambda }_{ij}$$

$$logP\left({Y}_{ij10}=1\right)={\lambda }_{ij}+\theta +{\alpha }_{i}+{\beta }_{j}$$

$$logP\left({Y}_{ij01}=1\right)={\lambda }_{ij}+\theta +{\alpha }_{j}+{\beta }_{i}$$

$$logP\left({Y}_{ij11}=1\right)={\lambda }_{ij}+2\theta +{\alpha }_{i}+{\alpha }_{j}+{\beta }_{i}+{\beta }_{j}+(\alpha \beta )$$

The $\left\{{\lambda }_{ij}\right\}$ parameters are mathematical necessities included in the model to ensure that the sum of these four probabilities equals one for each dyad. Thus these parameters appear in all four statements, regardless of the state of the dyad. The *θ* parameter is interpreted as an overall choice effect (analogous to a grand mean), reflecting the overall volume of choices sent and received. If one tie is reciprocated, two *θ*’s appear. Note that, *θ* does not appear in the model statement when ties are not present, and *(αβ)* is only present when the dyad is mutual. No substantive parameters appear in the first statement of the model which represents a null dyad. For asymmetric dyads, the log probabilities depend on parameters reflecting only one of the two possible ties in the dyad (i) dyads in which node *i* chooses node *j* without reciprocation (thus α_i_ is relevant but α_j_ is not and *β*_*j*_ is included but *β*_*i*_ is not) and (ii) dyads in which node *j* chooses node *i* with no reciprocated choice (thus the relevant parameters are α_j_ and *β*_*i*_, but not α_i_ or *β*_*j*_ or *(αβ)*. All the parameters appear together only for mutual dyads (the last statement of the model). The *(αβ) which is* sometimes denoted by *ρ*, is also called a mutuality parameter. The parameter will be positive and large when the relation tends to be mutual.

In the equations, the *α* parameters are interpreted as expansiveness measures for each node. If the *α* value of the corresponding node is positive and large, it can be concluded that there is a high probability that the node will influence the other nodes. The *β* parameters are interpreted as attractiveness measures. If the *β* value of the corresponding node is positive and large, it can be deduced that there is a high probability that the node is influenced by other nodes. The parameters are estimated using the principle of maximum likelihood method. In this study, only expansiveness and attractiveness parameters are considered for finding the relationships of the nodes.

## References

[CR1] Aertsen AM, Gerstein GL, Habib MK, Palm G (1989) Dynamics of neuronal firing correlation: modulation of “effective connectivity.” J Neurophysiol 61:900–917 2723733 10.1152/jn.1989.61.5.900

[CR2] Alexander SA (2013) Infinite graphs in systematic biology, with an application to the species problem. Acta Biotheor 61(2):181–201 23297024 10.1007/s10441-012-9168-y

[CR3] Badwaik JS (2020) Recent advances in graph theory and its applications. Int Res J Sci Eng Special Issue A7:533–538

[CR4] Bassett DS, Sporns O (2017) Network neuroscience. Nat Neurosci 20(3):353–364. 10.1038/nn.4502 28230844 10.1038/nn.4502 PMC5485642

[CR5] Boccaletti S, Latora V, Moreno Y, Chavez M, Hwang DU (2006) Complex networks: structure and dynamics. Phys Rep 424:175–308

[CR6] Bordier C, Nicolini C, Bifone A (2017) Graph analysis and modularity of brain functional connectivity networks: searching for the optimal threshold. Front Neurosci 11:441. 10.3389/fnins.2017.00441 28824364 10.3389/fnins.2017.00441 PMC5540956

[CR7] Brown EN, Kass RE, Mitra PP (2004) Multiple neural spike train data analysis: state-of-the-art and future challenges. Nat Neurosci 7:456–461 15114358 10.1038/nn1228

[CR8] Bruzzone SEP, Lumaca M, Brattico E, Vuust P, Kringelbach ML, Bonetti L (2022) Dissociated brain functional connectivity of fast versus slow frequencies underlying individual differences in fluid intelligence: a DTI and MEG study. Sci Rep 12:4746. 10.1038/s41598-022-08521-5 35304521 10.1038/s41598-022-08521-5 PMC8933399

[CR9] Buckner RL, Sepulcre J, Talukdar T, Krienen FM, Liu H, Hedden T, Johnson KA (2009) Cortical hubs revealed by intrinsic functional connectivity: mapping, assessment of stability, and relation to Alzheimer’s disease. J Neurosci 29(6):1860–1873. 10.1523/JNEUROSCI.5062-08.2009 19211893 10.1523/JNEUROSCI.5062-08.2009 PMC2750039

[CR84] Bullmore E, Sporns O (2009) Complex brain networks: graph theoretical analysis of structural and functional systems, Nature Reviews Neuroscience 10:186–198. 10.1038/nrn2575 19190637

[CR10] Buzsáki G (2006) Rhythms of the brain. Oxford University Press, Oxford

[CR11] Buzsáki G, Mizuseki K (2014) The log-dynamic brain: how skewed distributions affect network operations. Nat Rev Neurosci 15(4):264–278 24569488 10.1038/nrn3687 PMC4051294

[CR12] Chen Y, Lin SC, Zhou Y, Carmichael O, Müller HG, Wang JL (2024) Alzheimer’s Disease Neuroimaging Initiative, Gradient synchronization for multivariate functional data, with application to brain connectivity. J R Stat Soc Series B Stat Methodol 86(3):694–713. 10.1093/jrsssb/qkad140 39005888 10.1093/jrsssb/qkad140 PMC11239314

[CR13] Chiarion G, Sparacino L, Antonacci Y, Faes L, Mesin L (2023) Connectivity analysis in EEG data: a tutorial review of the state of the art and emerging trends. Bioengineering 10:372. 10.3390/bioengineering10030372 36978763 10.3390/bioengineering10030372 PMC10044923

[CR14] Cohen JR, D’Esposito M (2016) The segregation and integration of distinct brain networks and their relationship to cognition. J Neurosci 36(48):12083–12094. 10.1523/JNEUROSCI.2965-15.2016 27903719 10.1523/JNEUROSCI.2965-15.2016 PMC5148214

[CR15] Cohen AL, Fair DA, Dosenbach NUF, Miezin FM, Dierker D, Van Essen DC, Petersen SE (2008) Defining functional areas in individual human brains using resting functional connectivity MRI. Neuroimage 41(1):45–57. 10.1016/j.neuroimage.2008.01.066 18367410 10.1016/j.neuroimage.2008.01.066 PMC2705206

[CR16] De Abril IM, Yoshimoto J, Doya K (2018) Connectivity inference from neural recording data: Challenges, mathematical bases and research directions. Neural Netw 102:120–137 29571122 10.1016/j.neunet.2018.02.016

[CR17] De Vico Fallani F, Richiardi J, Chavez M, Achard S (2014) Graph analysis of functional brain networks: practical issues in translational neuroscience. Philos Trans R Soc Lond B Biol Sci 369:20130521. 10.1098/rstb.2013.0521 25180301 10.1098/rstb.2013.0521 PMC4150298

[CR18] Deco G, Hugues E (2012) Neural network mechanisms underlying stimulus driven variability reduction. PLoS Comput Biol 8(3):e1002395. 10.1371/journal.pcbi.1002395 22479168 10.1371/journal.pcbi.1002395 PMC3315452

[CR19] Dodel S, Hermann JM, Geisel T (2002) Functional connectivity by cross-correlation clustering. Neurocomputing 44–46:1065–1070

[CR20] Erciyes K (2023) Graph-theoretical analysis of biological networks: a survey. Computation 11(10):188. 10.3390/computation11100188

[CR21] Etkin A, Büchel C, Gross JJ (2015) The neural bases of emotion regulation. Nat Rev Neurosci 16(11):693–700. 10.1038/nrn4044 26481098 10.1038/nrn4044

[CR22] Fagiolo G (2007) Clustering in complex directed networks. J Phys Rev E Stat Nonlinear Soft Matter Phys 77:026107 10.1103/PhysRevE.76.026107 17930104

[CR23] Fang M, Poskanzer C, Anzellotti S (2023) Multivariate connectivity: a brief introduction and an open question. Front Neurosci 16:1082120. 10.3389/fnins.2022.1082120 36704006 10.3389/fnins.2022.1082120 PMC9871770

[CR24] Fiecas M, Ombao H, Linkletter C, Thompson W, Sanes J (2010) Functional connectivity: shrinkage estimation and randomization test. Neuroimage 49:3005–3014 20006714 10.1016/j.neuroimage.2009.12.022 PMC3128923

[CR25] Folias SE, Yu S, Snyder A, Nikolić D, Rubin JE (2013) Synchronisation hubs in the visual cortex may arise from strong rhythmic inhibition during gamma oscillations. Eur J Neurosci 38(6):2864–2883 23837724 10.1111/ejn.12287

[CR26] Freeman LC (1978) Centrality in social networks: conceptual clarification. Soc Netw 1:215–239

[CR27] Gerhard F, Pipa G, Lima B, Neuenschwander S, Gerstner W (2011) Extraction of network topology from multi-electrode recordings: is there a small-world effect? Front Comput Neurosci 5:1–13 21344015 10.3389/fncom.2011.00004 PMC3036953

[CR28] Guha S, Rodriguez-Acosta J, Dinov ID (2024) A bayesian multiplex graph classifier of functional brain connectivity across diverse tasks of cognitive control. Neuroinformatics 22(4):457–472. 10.1007/s12021-024-09670-w 38861097 10.1007/s12021-024-09670-w PMC11578796

[CR29] Hadley JA, Kraguljac NV, White DM, Hoef LV, Tabora J, Lahti AC (2016) Change in brain network topology as a function of treatment response in schizophrenia: a longitudinal resting-state fMRI study using graph theory. NPJ Schizophr. 10.1038/npjschz.2016.14 27336056 10.1038/npjschz.2016.14 PMC4898893

[CR30] Harary F, Palmer EM (1973) Graphical enumeration. Academic Press, New York, p 124

[CR31] Hasson U, Nir Y, Levy I, Fuhrmann G, Malach R (2004) Intersubject synchronization of cortical activity during natural vision. Science 303(5664):1634–1640. 10.1126/science.1089506 15016991 10.1126/science.1089506

[CR32] Hasson U, Landesman O, Knappmeyer B, Vallines I, Rubin N, Heeger DJ (2008) Neurocinematics: The neuroscience of film. Projections 2(1):1–26. 10.3167/proj.2008.020102

[CR33] Holland PW, Leinhardt S (1981) An exponential family of probability distributions for directed graphs. J Am Stat Assoc 76(373):33–50

[CR34] Hutchison RM, Womelsdorf T, Allen EA, Bandettini PA, Calhoun VD, Corbetta M, Chang C (2013) Dynamic functional connectivity: promise, issues, and interpretations. Neuroimage 80:360–378. 10.1016/j.neuroimage.2013.05.079 23707587 10.1016/j.neuroimage.2013.05.079 PMC3807588

[CR35] Islam MR, Yin X, Ulhaq A, Zhang Y, Wang H, Anjum N, Kron T (2017) A survey of graph based complex brain network analysis using functional and diffusional MRI. Am J Appl Sci 14(12):1186–1208. 10.3844/ajassp.2017.1186.1208

[CR36] Jafarzadeh N, Iranmanesh A (2016a) A new graph theoretical method for analyzing DNA sequences based on genetic codes. MATCH-Commun Math Comput Chem 75(3):731–742

[CR37] Jafarzadeh N, Iranmanesh A (2016b) Application of graph theory to biological problems. Studia Ubb Chemia LXI:9–16

[CR38] Jun JJ et al (2017) Fully integrated silicon probes for high-density recording of neural activity. Nature 551(7679):232–236 29120427 10.1038/nature24636 PMC5955206

[CR39] Kayser C, Petkov CI, Logothetis NK (2009) Multisensory interactions in primate auditory cortex: fMRI and electrophysiology. Hear Res 258(1–2):80–88. 10.1016/j.heares.2009.02.011 19269312 10.1016/j.heares.2009.02.011

[CR40] Kostelecki W, Dominguez LG, Perez Velazquez JL (2011) Single trial classification of magnetoencephalographic recordings using granger causality. J Neurosci Methods 199(2):183–91 21600926 10.1016/j.jneumeth.2011.04.032

[CR41] Latora V, Marchiori M (2001) Efficient behavior of small-world networks. Phys Rev Lett 87:198701 11690461 10.1103/PhysRevLett.87.198701

[CR42] Lee JH, Durand R, Gradinaru V, Zhang F, Goshen I, Kim DS, Fenno LE, Ramakrishnan C, Deisseroth K (2010) Global and local fMRI signals driven by neurons defined optogenetically by type and wiring. Nature 465(7299):788–792. 10.1038/nature09108 20473285 10.1038/nature09108 PMC3177305

[CR43] Liang J, Zhou C (2022) Criticality enhances the multilevel reliability of stimulus responses in cortical neural networks. PLoS Comput Biol 18(1):e1009848. 10.1371/journal.pcbi.1009848 35100254 10.1371/journal.pcbi.1009848 PMC8830719

[CR44] Liu J, Li M, Pan Y, Lan W, Zheng R, Wu FX, Wang J (2017) Complex brain network analysis and its applications to brain disorders: a survey. Complexity 2017:1–27. 10.1155/2017/8362741

[CR45] Lobov SA, Berdnikova ES, Zharinov AI, Kurganov DP, Kazantsev VB (2023) STDP-driven rewiring in spiking neural networks under stimulus-induced and spontaneous activity. Biomimetics 8:320. 10.3390/biomimetics8030320 37504208 10.3390/biomimetics8030320 PMC10807410

[CR46] Masud MS, Borisyuk R (2011) Statistical technique for analysing functional connectivity of multiple spike trains. J Neurosci Methods 196:201–219 21236298 10.1016/j.jneumeth.2011.01.003

[CR47] Mele G, Cavaliere C, Alfano V, Orsini M, Salvatore M, Aiello M (2019) Simultaneous EEG-fMRI for functional neurological assessment. Front Neurol 10:848. 10.3389/fneur.2019.00848. (**Aug 13;**) 31456735 10.3389/fneur.2019.00848 PMC6700249

[CR48] Mijalkov M, Kakaei E, Pereira JB, Westman E, Volpe G (2017) BRAPH: A graph theory software for the analysis of brain connectivity. PLoS ONE 12(8):e0178798. 10.1371/journal.pone.0178798 28763447 10.1371/journal.pone.0178798 PMC5538719

[CR49] Milo R, Shen-Orr S, Itzkovitz S, Kashtan N, Chklovskii D, Alon U (2002) Network motifs: simple building blocks of complex networks. Science 298:824–827 12399590 10.1126/science.298.5594.824

[CR50] Mullinger KJ, Cherukara MT, Buxton RB, Francis ST, Mayhew SD (2017) Post-stimulus fMRI and EEG responses: evidence for a neuronal origin hypothesised to be inhibitory. Neuroimage 157:388–399. 10.1016/j.neuroimage.2017.06.020 28610902 10.1016/j.neuroimage.2017.06.020 PMC6475192

[CR51] Nandagopal N, Elowitz MB (2011) Synthetic biology: integrated gene circuits. Science 333(6047):1244–1248 21885772 10.1126/science.1207084 PMC4117316

[CR52] Nikolic D (2007) Non-parametric detection of temporal order across pairwise measurements of time delays. J Comput Neurosci 22(1):5–19 16998643 10.1007/s10827-006-9441-7

[CR53] Nikolić D (2023) Where is the mind within the brain? Transient selection of subnetworks by metabotropic receptors and G protein-gated ion channels. Comput Biol Chem 103:107820 36724606 10.1016/j.compbiolchem.2023.107820

[CR54] Paninski L, Ahmadian Y, Ferreira DG, Koyama S, Rahnama Rad K, Vidne M, Vogelstein J, Wu W (2010) A new look at state-space models for neural data. J Comput Neurosci 29:107–126 19649698 10.1007/s10827-009-0179-x PMC3712521

[CR55] Perkel DH, Gerstein GL, Moore GP (1967) Neuronal spike trains and stochastic point processes. II. Simultaneous spike trains. Biophys J 7:419–440 4292792 10.1016/S0006-3495(67)86597-4 PMC1368069

[CR56] Pillow JW, Shlens J, Paninski L, Sher A, Litke AM, Chichilnisky EJ, Simoncelli EP (2008) Spatiotemporal correlations and visual signalling in a complete neuronal population. Nature 454:995–999 18650810 10.1038/nature07140 PMC2684455

[CR57] Pisarchik AN (2024) Computational and mathematical methods for neuroscience. Appl Sci 14(23):11296. 10.3390/app142311296

[CR58] Rajan RS, Anitha J, Rajasingh I (2015) 2-power domination in certain interconnection networks. Proc Comput Sci 57:738–744

[CR59] Ros T, Théberge J, Frewen PA, Kluetsch R, Densmore M, Calhoun VD, Lanius RA (2013) Mind over chatter: plastic up-regulation of the fMRI salience network directly after EEG neurofeedback. Neuroimage 65:324–335. 10.1016/j.neuroimage.2012.09.046 23022326 10.1016/j.neuroimage.2012.09.046 PMC5051955

[CR85] Rubinov M, Sporns O (2010) Complex network measures of brain connectivity: uses and interpretations, Neroimage 52:1059–1069. 10.1016/j.neuroimage.2009.10.003 19819337

[CR60] Saalmann YB, Pinsk MA, Wang L, Li X, Kastner S (2012) The pulvinar regulates information transmission between cortical areas based on attention demands. Science 337(6095):753–756. 10.1126/science.1223082 22879517 10.1126/science.1223082 PMC3714098

[CR61] Salvador R, Suckling J, Schwarzbauer C, Bullmore E (2005) Undirected graphs of frequency-dependent functional connectivity in whole brain networks. Philos Trans R Soc Lond B Biol Sci 360:937–946 16087438 10.1098/rstb.2005.1645 PMC1854928

[CR62] Schneider G, Havenith MN, Nikolic D (2006) Spatiotemporal structure in large neuronal networks detected from cross-correlation. Neural Comput 18:2387–2413 16907631 10.1162/neco.2006.18.10.2387

[CR63] Schneider DM, Sundararajan J, Mooney R (2018) A cortical filter that learns to suppress the acoustic consequences of movement. Nature 561(7724):391–395 30209396 10.1038/s41586-018-0520-5 PMC6203933

[CR64] Schneidman E, Berry MJ, Segev R, Bialek W (2006) Weak pairwise correlations imply strongly correlated network states in a neural population. Nature 440:1007–1012 16625187 10.1038/nature04701 PMC1785327

[CR65] Shamshiri EA, Sheybani L, Vulliemoz S (2019) The role of EEG-fMRI in studying cognitive network alterations in epilepsy. Front Neurol 10:1033. 10.3389/fneur.2019.01033 31608007 10.3389/fneur.2019.01033 PMC6771300

[CR66] Sitaram R, Ros T, Stoeckel L, Haller S, Scharnowski F, Lewis-Peacock J, Sulzer J (2017) Closed-loop brain training: the science of neurofeedback. Nat Rev Neurosci 18(2):86–100. 10.1038/nrn.2016.164 28003656 10.1038/nrn.2016.164

[CR67] Sonkusare S, Breakspear M, Guo C (2019) Naturalistic stimuli in neuroscience: critically acclaimed. Trends Cogn Sci 23(8):699–714. 10.1016/j.tics.2019.05.004 31257145 10.1016/j.tics.2019.05.004

[CR68] Sporns O (2002) Graph theory methods for the analysis of neural connectivity patterns. In: Kotter R (ed) Neuroscience databases: a practical guide. Klüwer, Boston, pp 171–186

[CR86] Sporns O (2010) Networks of the brain: quantitative analysis and modeling, Technical report, Department of Psychological and Brain Sciences, Indiana University, Bloomington, Indiana.

[CR69] Sporns O (2013) Structure and function of complex brain networks. Dialogues Clin Neurosci 15:247–262 24174898 10.31887/DCNS.2013.15.3/osporns PMC3811098

[CR70] Sporns O, Kotter R (2004) Motifs in brain networks. PLoS Biol 2:1910–1918 10.1371/journal.pbio.0020369 PMC524253 15510229

[CR71] Sporns O, Christopher JH, Kotter R (2007) Identification and classification of hubs in brain networks. PLoS ONE 2(10):e1049 17940613 10.1371/journal.pone.0001049 PMC2013941

[CR72] Stam CJ (2004) Functional connectivity patterns of human magnetoencephalographic recordings: a “small-world” network? Neuroscience Letter 355:25–28 10.1016/j.neulet.2003.10.063 14729226

[CR73] Stam CJ, Jones BF, Nolte G, Breakspear M, Scheltens P (2007) Small world networks and functional connectivity in Alzheimer’s disease. Cereb Cortex 17:92–99 16452642 10.1093/cercor/bhj127

[CR74] Stevenson IH, Kording KP (2011) How advances in neural recording affect data analysis. Nat Neurosci 14(2):139–142 21270781 10.1038/nn.2731 PMC3410539

[CR75] Tanamachi K, Kuwahara W, Okawada M, Sasaki S, Kaneko F (2023) Relationship between resting-state functional connectivity and change in motor function after motor imagery intervention in patients with stroke: a scoping review. J Neuro Eng Rehabil 20:159. 10.1186/s12984-023-01282-w 10.1186/s12984-023-01282-w PMC10657492 37980496

[CR76] Truccolo W, Eden UT, Fellows MR, Donoghue JP, Brown EN (2005) A point process framework for relating neural spiking activity to spiking history, neural ensemble, and extrinsic covariate effects. J Neurophysiol 93:1074–1089 15356183 10.1152/jn.00697.2004

[CR77] Vecchio F, Miraglia F, Rossini PM (2017) Connectome: graph theory application in functional brain network architecture. Clin Neurophysiol Pract 2:206–213 30214997 10.1016/j.cnp.2017.09.003 PMC6123924

[CR78] Warbrick T (2022) Simultaneous EEG-fMRI: what havewe learned and what does the future hold? Sensors 22:2262. 10.3390/s22062262 35336434 10.3390/s22062262 PMC8952790

[CR79] Watts DJ, Strogatz SH (1998) Collective dynamics of “small-world” networks. Nature 393:440–442 9623998 10.1038/30918

[CR80] Wu J, Kendrick K, Feng J (2007) Detecting correlation changes in electrophysiological data. J Neurosci Methods 161:155–165 17137633 10.1016/j.jneumeth.2006.10.017

[CR81] Yger P et al (2018) A spike sorting toolbox for up to thousands of electrodes validated with ground truth recordings *in vitro* and *in vivo*. Elife 7:e34518 29557782 10.7554/eLife.34518 PMC5897014

[CR82] Young MP (1993) The organization of neural systems in the primate cerebral cortex. Proc R Soc Lond B 252(1993):13–18 10.1098/rspb.1993.0040 8389046

[CR83] Yu S, Huang D, Singer W, Nikolić D (2008) A small world of neuronal synchrony. Cereb Cortex 18(12):2891–2901 18400792 10.1093/cercor/bhn047 PMC2583154

